# Twelve new species of *Dipara* Walker, 1833 (Hymenoptera, Chalcidoidea, Pteromalidae, Diparinae) from Kenya, with a key to the Afrotropical species

**DOI:** 10.3897/zookeys.1067.72395

**Published:** 2021-10-29

**Authors:** Christoph Braun, Ralph S. Peters

**Affiliations:** 1 Leibniz Institute for the Analysis of Biodiversity Change, Zoological Research Museum Alexander Koenig, Adenauerallee 160, 53113 Bonn, Germany Zoological Research Museum Alexander Koenig Bonn Germany

**Keywords:** Morphometry, parasitoid wasps, taxonomy

## Abstract

Based on 261 female specimens of the genus *Dipara* Walker, 1833 from leaf litter samples of the Kakamega Forest in Kenya, we describe the following twelve new species: *Diparaandreabalzerae***sp. nov.**, *Diparacorona***sp. nov.**, *Diparafastigata***sp. nov.**, *Diparakakamegensis***sp. nov.**, *Diparalux***sp. nov.**, *Diparanigroscutellata***sp. nov.**, *Diparanyani***sp. nov.**, *Diparareticulata***sp. nov.**, *Dipararodneymulleni***sp. nov.**, *Diparasapphirus***sp. nov.**, *Diparatenebra***sp. nov.**, and *Diparatigrina***sp. nov.** For *Diparaalbomaculata* (Hedqvist, 1963) and *Diparanigrita* Hedqvist, 1969, we give new distribution records. We examined the available type material of all described *Dipara* species from the Afrotropical mainland, i.e., *Diparaalbomaculata* (Hedqvist, 1963), *Diparamachadoi* (Hedqvist, 1971), *Diparamaculata* (Hedqvist, 1963), *Diparanigrita* Hedqvist, 1969, *Diparapallida* (Hedqvist, 1969), *Diparapunctulata* (Hedqvist, 1969), *Diparasaetosa* (Delucchi, 1962), *Diparastraminea* (Hedqvist, 1969), *Diparastriata* (Hedqvist, 1969), and *Diparaturneri* Hedqvist, 1969. We provide figures, descriptions, and diagnoses of the newly described species and figures and diagnoses of the ten known species as well as an identification key to all species of the Afrotropical mainland.

## Introduction

In this study, we contribute to the taxonomic knowledge of the Afrotropical fauna of the genus *Dipara* Walker, 1833, with the first alpha-taxonomic treatment of this group and region in 50 years. We describe twelve new species and diagnose and key all new and previously described Afrotropical mainland species. *Dipara* belongs to the subfamily Diparinae within the chalcidoid family Pteromalidae (Heraty et al. 2012). The genus shows a cosmopolitan distribution (Desjardins 2007) with a total of 56 described species (Noyes 2019). The phylogenetic position of Diparinae is still unclear (Desjardins 2007; Heraty et al. 2012). Currently, it is still classified within Pteromalidae, which, however, is polyphyletic (Peters et al. 2018). Diparinae were shown to be monophyletic and can be identified by the following diagnostic characters: presence of a cercal brush and absence of a smooth convex dorsellum (Desjardins 2007). The genus *Dipara* is well characterized by a number of diagnostic characters (see below or Desjardins 2007 for a full diagnosis and a list of genera previously synonymized under *Dipara*).

The early taxonomic work on *Dipara* was confounded by the strong sexual dimorphism in this group. Males are usually macropterous and have filiform antennae. Females can range from macropterous to apterous and have clavate antennae. Additionally, males tend to be extremely similar even across different genera while females show a lot of interspecific morphological variation (Desjardins 2007). This led to the genus originally being described by Walker (1833) based on a male specimen of *Diparapetiolata* Walker, 1833 and *Dipara* females originally being described as *Tricoryphus* by Förster (1856) and as *Hispanolelaps* by Mercet (1927). The two genera were later synonymized with *Dipara* by Domenichini (1953). Because of the strong resemblance of males of different species and the morphological variation of females, most species level taxonomic work on *Dipara* (and other Diparinae) is based on female specimens (Delucchi 1962; Hedqvist 1963, 1969, 1971). For males, Desjardins (2007) provided a genus level key. Matching females and males of the same species based on morphological features is currently not possible. Mitroiu (2019) suggested to match conspecific females and males based on molecular sequence data (e.g., the DNA barcode) and this is certainly the way to go. Unfortunately, the material available for this study was not suitable for standard DNA sequencing and consistently failed in a pre-study trial (unpublished). Accordingly, our work is based solely on morphological characters of females.

A peculiar characteristic of Diparinae females is their intraspecific variation in the wing form with macropterous and brachypterous specimens being found in the same species (Bouček 1988; Desjardins 2007; Mitroiu 2019). To deal with this potentially confounding fact, we used a multivariate morphometric approach (Baur and Leuenberger 2011) in morphologically similar species with different wing forms, which has been applied successfully numerous times for taxonomic studies on parasitoid wasps (e.g., László et al. 2013; Baur et al. 2014; Baur 2015; Gebiola et al. 2017; Werner and Peters 2018). Additionally, we checked the state of the posterior notal wing process which Desjardins (2007) suggested to be a “measure of potential wing size”, i.e., a possible hint on the intraspecific wing form variation.

There is a severe lack of information about the biology of *Dipara* species. One of their main habitats is supposed to be leaf litter (Desjardins 2007). The only published information about their hosts is that of an unidentified Indian *Dipara* species which was reared from a curculionid beetle feeding on the roots of a *Cyperus* species (Bouček 1988). Additional host records from curculionids in *Lelaps* Walker, 1843 led Desjardins (2007) to suggest that the more common and typical Diparinae (like *Lelaps* and *Dipara* species) may parasitize soil-inhabiting beetles and maybe curculionids more specifically.

So far, ten species of *Dipara* have been described from the Afrotropical mainland, with a distribution ranging from the Democratic Republic of Congo to South Africa, including *Diparaalbomaculata* (Hedqvist, 1963), *Diparamachadoi* (Hedqvist, 1971), *Diparamaculata* (Hedqvist, 1963), *Diparanigrita* Hedqvist, 1969, *Diparapallida* (Hedqvist, 1969), *Diparapunctulata* (Hedqvist, 1969), *Diparasaetosa* (Delucchi, 1962), *Diparastraminea* (Hedqvist, 1969), *Diparastriata* (Hedqvist, 1969) and *Diparaturneri* Hedqvist, 1969 (Mitroiu 2011; Noyes 2019). Larger series of *Dipara* specimens are exceedingly rare and descriptions are often based on a single or just a few specimens. Five Afrotropical *Dipara* species are known only from the holotype and nine from five specimens or less. Only *D.pallida* is known from a larger series of 13 specimens (Desjardins 2007).

We based our work on an extraordinary series of 261 female *Dipara* specimens from the Kakamega Forest in Kenya. Collection of the specimens was done in the framework of the BIOTA (BIOdiversity Monitoring Transect Analysis in Africa) East Africa project (Ross et al. 2018). The Kakamega Forest is a montane rainforest fragment in western Kenya and the easternmost remnant of the Guineo-Congolian rainforest belt (Kokwaro 1988; Clausnitzer 2005; Holstein 2015). Due to high rural population density around the Kakamega Forest it is under high threat from deforestation and habitat destruction (KIFCON 1994). Parts of its plant and animal fauna have already been studied in detail (e.g., Althof 2005; Clausnitzer 2005; Kühne 2008; Hita-Garcia et al. 2013). To preserve biodiversity, it is a most urgent and necessary task to contribute to the knowledge of highly diverse, threatened habitats, including knowledge on parasitoid wasps of these areas, by increasing visibility of species from this region and making specimens from it available.

With the description of twelve new *Dipara* species from the leaf litter of Kakamega Forest in Kenya we can show that the species diversity of the genus has not been sufficiently studied and the true diversity of Afrotropical *Dipara*, and presumably other Diparinae, has been underestimated. Since our very much geographically limited study already more than doubles the number of known species, we expect that numerous additional species of Afrotropical *Dipara* still await discovery and description. This study may serve as a starting point for future in-depth investigations, including thorough taxonomic revisions of the Afrotropical Diparinae, Chalcidoidea or, more generally, parasitoid wasp fauna.

## Materials and methods

In the following, abbreviations are given of the museums where the material used in this study is stored. The abbreviations will be used throughout the text.

**MDLA** Laboratório de Biologia, Dundo, Lunda, Angola

**NHMUK** Natural History Museum, London, UK

**NMK** National Museums of Kenya, Nairobi

**RMCA** Royal Museum for Central Africa, Tervuren, Belgium

**ZFMK** Zoologisches Forschungsmuseum Alexander Koenig, Bonn, Germany

The terminology is based on Gibson (1997) and the Hymenoptera Anatomy Ontology portal (Yoder et al. 2010). For the terminology of the surface sculpture, we used Harris (1979).

A total of 261 female *Dipara* specimens from the Kakamega Forest in Kenya were examined. They were collected in 2007 and 2008 using Winkler extraction of a 1 m² leaf litter sample in multiple transects throughout the Kakamega Forest (Ross et al. 2018) and stored in 70% ethanol at room temperature at the ZFMK. All female *Dipara* specimens were isolated from the collective leaf litter samples and transferred to 99.8% ethanol. After presorting and examination, the specimens were critical point dried using a Leica EM CPD 300 AUTO and mounted on small, pointed cardboard plates with shellac-based glue. Morphological examinations were done with a Zeiss Discovery V8 stereomicroscope with a Plan S 1.0× FWD 81mm objective and PI 10×/23 eyepieces.

Digital imaging was done with a Keyence VHX-2000 digital microscope. For images of the dorsal and lateral habitus and the head the VHX-J250 objective (250–2500×) was used. The images were stacked and edited in brightness, coloration and contrast using the Keyence internal software. Further editing of figures was done with Microsoft Power Point 2010. For the images for the morphometric measurements the Keyence VH-Z20R objective (20–200×) with a magnification of 200× was used. For the body length and the gaster length magnifications of 100× or 150× were used if the character did not fit into an image with 200× magnification. After calibration, measurements were done using ImageJ 1.53a. Characters used for morphometric measurements are given and explained in Table 1.

**Table 1.** List of morphometric characters with abbreviations and definitions (character definitions are based on Graham (1969), Gibson (1997) and Baur (2015)). Characters highlighted in bold were used for the morphometric analysis of *D.kakamegensis* sp. nov. and *D.nyani* sp. nov. (see Tables S1 and S2 for results).

Abbr.	Character	Definition
**scp.l**	Scape length	Length of scape exclusive of radicle, outer aspect
**pdl.l**	Pedicel length	Length of pedicel, outer aspect
**pdl.b**	Pedicel breadth	Breadth of pedicel, outer aspect
**pdl.flg**	Pedicel + flagellum	Combined length of pedicel plus flagellum, outer aspect
**clv.l**	Clava length	Length of clava, outer aspect
**clv.b**	Clava breadth	Breadth of clava, outer aspect
tor.d	Toruli diameter	Greatest diameter of right torulus, outer aspect
ant.d	Antennae distance	Greatest distance between outer edges of toruli
ant.eye	Distance from antennal insertion to eye	Distance between center of insertion point of antennae and level of ventral margin of the eyes measured straight down from insertion point of antennae
**eye.b**	Eye breadth	Greatest breadth of eye, lateral view
**eye.h**	Eye height	Greatest length of eye height, lateral view
**mspl.l**	Malar space length	Distance between the point where malar sulcus enters mouth margin and malar sulcus enters lower edge of eye, lateral view
**hea.h**	Head height	Distance between lower edge of clypeus and lower edge of anterior ocellus, frontal view
**upf.l**	Upper face length	Distance between lower edge of toruli and lower edge of anterior ocellus
**hea.b**	Head breadth	Greatest breadth of head, dorsal view
**eye.d**	Eye distance	Shortest distance between eyes, dorsal view
**pol.l**	POL	Shortest distance between posterior ocelli, dorsal view
**ool.l**	OOL	Shortest distance between posterior ocellus and eye margin, dorsal view
**prn.l**	Pronotum length	Length of pronotum along median line from anterior edge of pronotum collar to anterior edge of mesoscutum
prn.b	Pronotum breadth	Greatest breadth of pronotum, dorsal view
**msc.b**	Mesoscutum breadth	Greatest breadth of mesoscutum just in front of level of tegula, dorsal view
**msc.l**	Mesoscutum length	Length of mesoscutum along median line from posterior edge of pronotum to posterior edge of mesoscutum, dorsal view
**mss.l**	Mesosoma length	Length of mesosoma along median line from anterior edge of pronotum collar to posterior edge of nucha, dorsal view
**sctl.l**	Mesoscutellum length	Length of mesoscutellum along median line from posterior edge of mesoscutum to posterior edge of mesoscutellum, dorsal view
**ppd.l**	Propodeum length	Length of propodeum measured along median line from anterior edge to posterior edge of nucha, dorsal view
**fm3.l**	Metafemur length	Length of metafemur, from distal end of trochanter to tip of metafemur, measured along midline, outer aspect
**fm3.b**	Metafemur breadth	Greatest breadth of metafemur, outer aspect
**ptl.l**	Petiole length	Length of petiole measured along median line, from posterior edge of nucha to posterior edge of petiole, dorsal view
ptl.b	Petiole breadth	Greatest breadth of petiole, outer aspect, dorsal view
**gst.l**	Gaster length	Length of gaster along median line from posterior edge of nucha to tip of ovipositor sheath, dorsal view

The range of the morphometric measurements is given in the species description with the value for the holotype in parentheses. If more than five specimens were present, five specimens were used for the morphometric measurements, and their respective collection numbers are given in parentheses at the beginning of the taxonomic treatment (see below). If five or less specimens were available, all specimens were used. In a few cases the number of specimens used for a certain measurement varies from the total number of specimens used, either because the measured character was not visible in some specimens or because more specimens were used for the in-depth morphometric analysis, using a subset of the characters (see below). In these cases, the collection numbers of specimens used are given in parentheses directly after the respective measurement (see Suppl. material 1: Table S1 and Suppl. material 2: Table S2). Some morphometric characters were used to calculate ratios. For these ratios different categories were defined to simplify the description of shape (Table 2). The shape ratios are given in Suppl. material 3: Table S3.

**Table 2.** Shape categories for morphometric measurements and ratios in the species descriptions.

Character	Categories			
Body length (in µm)	**small**	**medium**	**large**	
	< 2000	2000–3000	> 3000	
Head shape in frontal view (head breadth/head height)	**round**		**oval**	
	< 1.31		> 1.31	
Mesosoma shape in dorsal view (head breadth/mesoscutum breadth)	**robust**	**of medium breadth**	**slender**	
	< 1.20	1.20–1.50	> 1.50	
Antennae distance (antennae distance/torulus diameter)	**close**		**far apart**	
	< 1.33		> 1.33	
Distance from antennal insertion to eye (distance from antennal insertion to eye/torulus diameter)	**same level as eyes**	**short**	**long**	
	0	< 1.1	> 1.1	
Pronotum shape in dorsal view (pronotum breadth/pronotum length)	**large and elongated**	**of medium length**	**short and slim**	
	< 2.5	2.5–3.5	> 3.5	
Petiole length (petiole length/petiole breadth)	**short**	**medium**	**long**	**very long**
	< 1.5	1.5–2.0	2.0–2.5	> 2.5
Gaster length (gaster length/mesosoma length)	**short**	**medium**	**long**	
	< 1.20	1.20–2.0	> 2.0	

### Morphometric analysis

Two putative species were found that clearly differed in the wing form, with one being macropterous and the other being brachypterous, but they were otherwise very similar with no obvious qualitative characters found to separate them. Since wing form might vary within species (Bouček 1988; Desjardins 2007; Mitroiu 2019), we chose to apply a quantitative approach based on multivariate morphometric analysis (Baur and Leuenberger 2011, 2020; Baur et al. 2015). For this purpose, the characters highlighted in bold in Table 1 were imaged and measured, as explained before, for 30 specimens of the first species (that later became *D.kakamegensis* sp. nov., see below) and all five specimens of the second species (that later became *D.nyani* sp. nov., see below). The measurements (given in Suppl. material 1: Table S1) were subsequently analyzed using R 4.0.2 and the R script package by Baur and Leuenberger (2020). Missing data was added using the imputation function of the mice R package.

### Posterior notal wing process

As suggested by Desjardins (2007), the posterior notal wing process (pnwp) can be used as a “measure of potential wing size”. The pnwp can be absent in brachypterous or apterous species, leading to the assumption that a fully developed pnwp in a brachypterous species could mean that macropterous individuals of this species exist. Desjardins (2007) lists four different character states: present and pointed, present but squarely truncate, present but truncate and rounded, and absent. We examined the state of the pnwp in each new species and imaged specimens of four different species with varying wing forms, from brachypterous to macropterous (Fig. 4), using the Keyence with the VHXJ-250 objective as described above.

## Results

### Morphometric analysis

**Figure 1.**
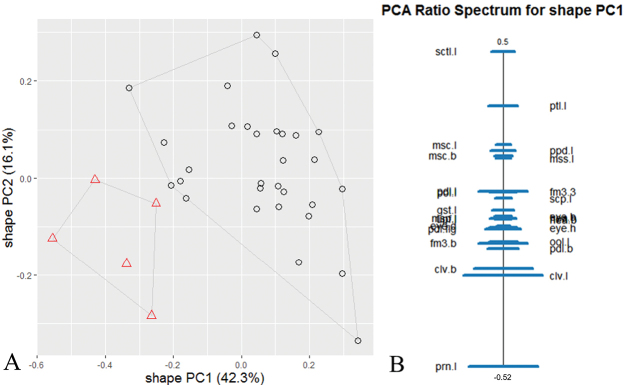
Shape analysis of *D.kakamegensis* sp. nov. (circles) and *D.nyani* sp. nov. (triangles) using the characters highlighted in bold in Table 1**A** scatterplot of first against second shape PC**B** ratio spectrum of the first shape PC; horizontal bars represent 68% confidence intervals based on 1000 bootstrap replicates.

The morphometric analysis of specimens of the two morphologically similar putative species showed that they can be reliably separated (Fig. 1 and 3). In the following, they will be treated as *D.kakamegensis* sp. nov. and *D.nyani* sp. nov.

**Figure 2.**
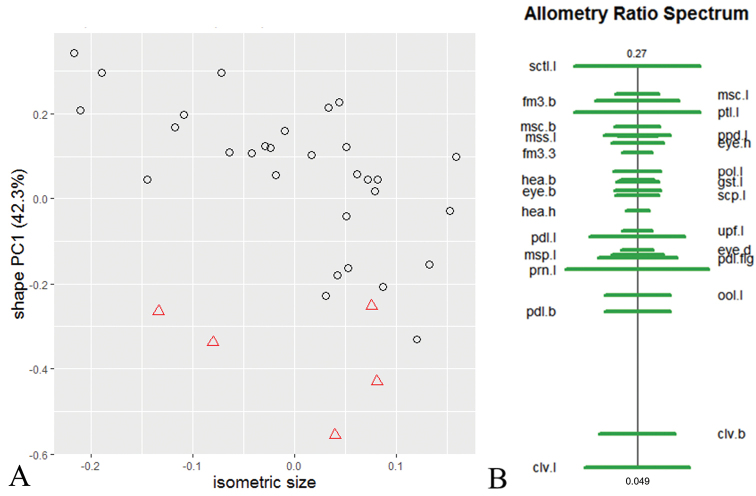
Allometry analysis of *D.kakamegensis* sp. nov. (circles) and *D.nyani* sp. nov. (triangles) **A** scatterplot of the isometric size against the first shape PC**B** allometry ratio spectrum; horizontal bars represent 68% confidence intervals based on 1000 bootstrap replicates.

Based on the results of the scree graph (not shown), only the first and second principal component (PC) were relevant for the further analysis of shape. The results of the shape PCA (Fig. 1A) of the two species show that they are separated by shape. The ratio spectrum (Fig. 1B) shows which ratios had the highest impact on the first shape PC. To confirm that these differences are based on true shape differences and not allometric size effects, the isometric size was plotted against the first shape PC (Fig. 2A). The species overlap in size but lie on different allometry axis, confirming that the separation is based on shape and not on allometry effects.

**Figure 3.**
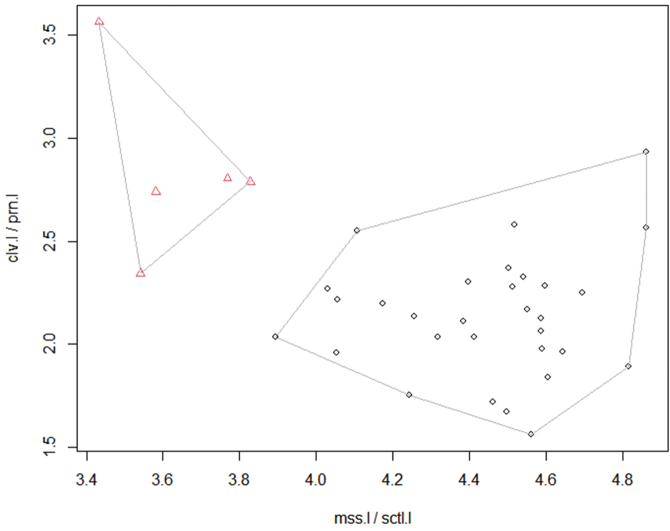
Scatterplot of mss.l/sctl.l against clv.l/prn.l, the ratios which separate the two species the best (based on the LDA ratio extractor); *D.kakamegensis* sp. nov. (circles) and *D.nyani* sp. nov. (triangles).

The LDA ratio extractor (Baur and Leuenberger 2020) found the best ratios to separate the two species: mss.l/sctl.l was the best ratio, clv.l/prn.l was the second best ratio (Fig. 3).

The allometry ratio spectrum (Fig. 2B) reveals the allometric variation of ratios. The characters of the best ratio (mss.l/sctl.l) lie close to each other, indicating no strong allometric effects. The characters of the second-best ratio (clv.l/prn.l) show a higher allometric effect than the first one but still not a considerable one. This confirms that the differences in these characters are based on shape and not on allometric effects.

The separating ratios were used for the diagnoses of the two species in the descriptions below.

### Posterior notal wing process

**Figure 4.**
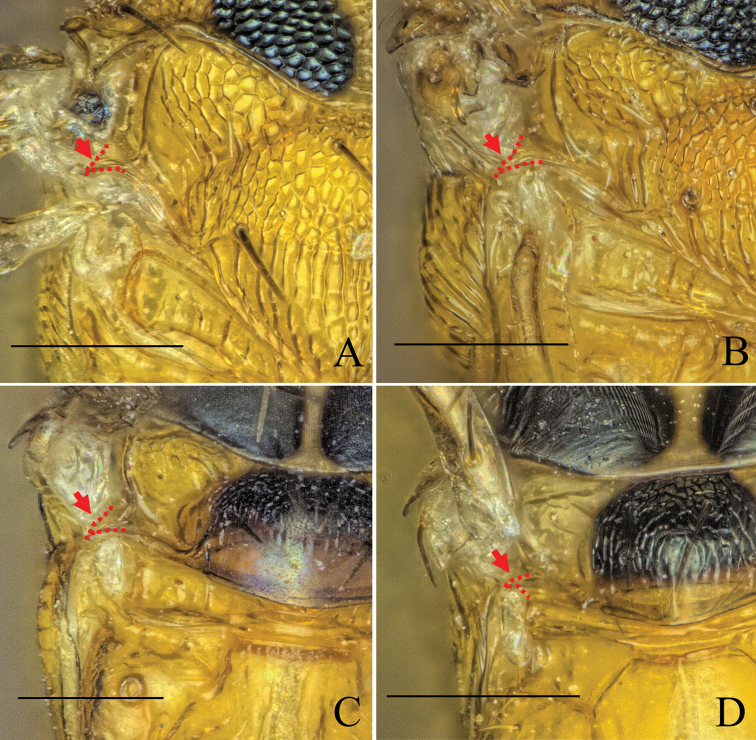
Dorsal view of a part of the mesosoma showing the posterior notal wing process (red) of **A***D.nyani* sp. nov. (macropterous) **B***D.kakamegensis* sp. nov. (brachypterous with medium sized wings), **C***D.andreabalzerae* sp. nov. (brachypterous with medium sized wings) and **D***D.nigroscutellata* sp. nov. (brachypterous with small wings). The former two are very similar but can be separated by morphometric analysis. Scale bar: 100 µm.

Examination of the posterior notal wing process (pnwp) in the newly described species showed that it was present and pointed in all cases. Figure 4 shows a selection of pnwps from species with different wing forms. The uniformity of this character can be interpreted as it either being unsuitable as a measure of potential wing size (see Desjardins 2007) or as indicating that all species, including the brachypterous ones, harbor also macropterous specimens. Accordingly, it proved little help in delimiting species, especially in the case of the very similar, but morphometrically discriminated macropterous *D.nyani* sp. nov. and brachypterous *D.kakamegensis* sp. nov. (see morphometric analysis above and taxonomic treatment below).

### Taxonomic treatments

#### Dipara

Taxon classificationAnimaliaHymenopteraPteromalidae

Walker, 1833 (modified from Desjardins 2007)

6C15A38C-04E0-5114-8297-88B12040D762

Dipara Walker 1833: 371, 373. Type species: Diparapetiolata Walker (by monotypy). Type locality: NHMUK.
Tricoryphus Förster 1856. Type Species: Tricoryphusfasciatus Thomson (by subsequent monotypy (Thomson 1876)). [Synonymized by Domenichini 1953]
Apterolelaps Ashmead 1901. Type Species: Apterolelapsnigriceps Ashmead (orig. desig. and by monotypy). [Synonymized by Graham 1969]
Alloterra Kieffer and Marshall 1904: 46–47. Type species: Alloterraclaviger Kieffer and Marshall (by monotypy). [Synonymized by Desjardins 2007]
Trimicrops Kieffer 1906. Type Species: Trimicropsclaviger Kieffer (by monotypy). [Synonymized by Desjardins 2007]
Parurios Girault 1913: 318. Type species: Paruriosaustraliana Girault (by monotypy). [Synonymized by Desjardins 2007]
Epilelaps Girault 1915: 344. Type species Epilelapshyalinipennis Girault (orig. desig.). [Synonymized by Bouček 1988]
Pseudipara Girault 1915: 345. Type species: Pseudiparaalbiclava Girault (orig. desig. and by monotypy). [Synonymized by Desjardins 2007]
Uriolelaps Girault 1915: 201. Type species: Uriolelapsargenticoxae Girault (orig. desig.). [Synonymized by Desjardins 2007]
Hispanolelaps Mercet 1927: 49–63. [Synonymized by Domenichini 1953]
Pseudiparella Girault 1927: 334–335. Type species Pseudiparellaemersoni Girault (by monotypy). [Synonymized by Bouček 1988]
Emersonia Girault 1933: [1]. Type species: Emersoniaatriscutum Girault (by monotypy). [Synonymized by Desjardins 2007]
Grahamisia Delucchi 1962: 379–380. Type species: Grahamisiasaetosa Delucchi (orig. desig. and by monotypy). [Synonymized by Desjardins 2007]
Afrolelaps Hedqvist 1963: 47. Type species: Afrolelapsmaculata Hedqvist (orig. desig.). [Synonymized by Desjardins 2007]
Pondia Hedqvist 1969: 197. Type species: Pondiapunctulata Hedqvist (orig. desig.). [Synonymized by Desjardins 2007]
Diparomorpha Hedqvist 1971: 57–58. Type species: Diparomorphamachadoi Hedqvist (orig. desig. and by monotypy). [Synonymized by Desjardins 2007]

##### Diagnosis.

**Female** (taken from Desjardins 2007). Absence of a median clypeal tooth; anellus broader than long; at most two pairs of mesoscutellar bristles; at least one pair of setae or bristles laterally on the petiole.

##### Remarks.

The Diparinae genus key by Desjardins (2007) states that the petiole of females of the genus *Dipara* is usually less than 1.5× as long as wide. In the present study, seven out of twelve newly described species have a petiole length exceeding this, going up to being 2.8× as long as wide. Accordingly, the genus level key of Desjardins (2007) might be misleading. However, the diagnosis by Desjardins (2007) uses other characters than the petiole length and can be kept unmodified (see above).

### Key to the *Dipara* species from the Afrotropical mainland (females)

**Table:** 

1	Notauli present (Figs 5–25)	**2**
–	Notauli absent	***D.machadoi* (Hedqvist, 1971)**
2(1)	Petiole with thicker and longer bristle anterio-laterally, reaching gt1 (Figs 20B, 21D)	**3**
–	Petiole with one or multiple thin and shorter setae laterally	**4**
3(2)	Vertex and propodeum smooth (Fig. 20)	***D.pallida* (Hedqvist, 1969)**
–	Vertex reticulate; propodeum subcarinate (Fig. 21)	***D.punctulata* (Hedqvist, 1969)**
4(2)	Head and mesosoma never completely black, usually dark brown or lighter, sometimes bright yellowish brown; if head and mesosoma partly dark brown to black, then pro- and metacoxa white (Fig. 17A)	**5**
–	Head and mesosoma black; coxae dark brown (Figs 18, 19)	***D.nigrita* Hedqvist, 1969**
5(4)	Face with one or two transverse dark brown to black stripes, reaching from one eye to the other, sometimes interrupted or fainter in interantennal and supraclypeal area (Figs 6B, 8B, 9B, 11B, 12B, 13B, 15B, 16B, 25B)	**6**
–	Face without distinct dark stripes, uniformly colored or sometimes with diffuse darker coloration (Figs 5B, 7B, 10B, 14B)	**16**
6(5)	Face with one transverse dark brown to black stripe, reaching from one eye to the other at the level of the ventral margin of the eyes (Figs 6B, 9B, 15B, 16B, 25B)	**7**
–	Face with two transverse dark brown to black stripes at the level of toruli and at the level of the ventral margin of the eyes, enclosing a stripe of brighter coloration (Figs 8B, 11B, 12B, 13B)	**12**
7(6)	Median and lateral area of mesoscutum with distinct transverse broad black stripe (Figs 6C, 25C)	**8**
–	Lateral areas of mesoscutum with two black spots (Figs 9C, 15C, 16C, 24C)	**9**
8(7)	Macropterous, fore wings reaching gt7; petiole distinctly longer than wide (Fig. 6C)	***D.corona* sp. nov.**
–	Brachypterous, fore wings reaching slightly beyond petiole; petiole slightly wider than long (Fig. 25C)	***D.turneri* Hedqvist, 1969**
9(7)	Petiole short, < 1.5× as long as wide (Figs 16D, 24C)	**10**
–	Petiole very long, > 2.5× as long as wide (Figs 9C, 15C)	**11**
10(9)	Propodeum medially reticulated between carinae (Fig. 16C); petiole with at least six pairs of small setae laterally (visible in dorsal view) (Fig. 16 D)	***D.tigrina* sp. nov.**
–	Propodeum without reticulation between carinae; petiole with three pairs of small setae laterally (visible in dorsal view) (Fig. 24C)	***D.striata* (Hedqvist, 1969)**
11(9)	Body brown to dark brown; vertex smooth (Fig. 15)	***D.tenebra* sp. nov.**
–	Body yellowish brown; vertex reticulate (Fig. 9)	***D.lux* sp. nov.**
12(6)	Gastral tergites smooth (Fig. 8C, 11C, 13C)	**13**
–	Gastral tergites reticulate (Fig. 12C)	***D.reticulata* sp. nov.**
13(12)	Lateral area of mesoscutum with two black spots; petiole long, < 2.1× as long as wide (Figs 8C, 11C)	**14**
–	Mesoscutum without distinct black coloration; petiole very long, 2.53–2.80× as long as wide (Fig. 13C)	***D.rodneymulleni* sp. nov.**
14(13)	Mesocoxa and petiole bright yellowish brown (Figs 8, 11)	**15**
–	Mesocoxa and petiole white	***D.maculata* (Hedqvist, 1963)**
15(14)	Brachypterous, fore wing reaching middle of gt1; mesoscutellum smaller, mesosoma length 3.90–4.86× mesoscutellum length (Fig. 8); petiole shorter, 1.15–1.72× as long as wide in dorsal view	***D.kakamegensis* sp. nov.**
–	Macropterous, fore wings reaching gt7; mesoscutellum larger, mesosoma length 3.43–3.83× mesoscutellum length (Fig. 11); petiole longer, 1.78–2.05× as long as wide in dorsal view	***D.nyani* sp. nov.**
16(5)	Mesoscutellum black (Figs 5C, 7C, 10C, 17B, 22C)	**17**
–	Mesoscutellum not black (14C, 23C)	**21**
17(16)	Propodeum medially smooth and laterally transversely carinate; gt1 without bristles (Figs 12C, 13C)	**18**
–	Propodeum completely smooth; gt1 with a pair of large bristles dorso-anteriorly (Figs 10C, 22C)	**19**
18(17)	Gaster brown to yellowish brown; anterior part of mesoscutellum and frenum forming an angle of 120–125° in lateral view (Fig. 5A)	***D.andreabalzerae* sp. nov.**
–	Gaster dark brown; anterior part of mesoscutellum and frenum forming an angle of 90° in lateral view (Fig. 7A)	***D.fastigata* sp. nov.**
19(17)	Body brown to dark brown; lateral area of mesoscutum completely black (Figs 17, 22)	**20**
–	Body yellowish brown to brown; lateral area of mesoscutum not completely black with small yellowish brown area on its most lateral part (Fig. 10C)	***D.nigroscutellata* sp. nov.**
20(19)	Vertex smooth; clava white; pro- and metacoxa white (Fig. 17)	***D.albomaculata* (Hedqvist, 1963)**
–	Vertex reticulate between ocelli, rest smooth; clava dark brown; pro- and metacoxa with proximal 1/3 brown and rest yellowish brown (Fig. 22)	***D.saetosa* (Delucchi, 1962)**
21(16)	Only slight metallic tint on black parts of the mesoscutum; lateral areas of mesoscutum with two black spots; gt1 with a pair of large bristles dorso-anteriorly (Fig. 23 C)	***D.straminea* (Hedqvist, 1969)**
–	Strong blue metallic tint on the following areas: vertex between ocelli, pronotum laterally, median area of mesoscutum posteriorly between notauli, lateral area of mesoscutum, mesoscutellum; lateral areas of mesoscutum without black spots; gt1 without a pair of large bristles dorso-anteriorly (Fig. 14C)	***D.sapphirus* sp. nov.**

#### Dipara andreabalzerae sp. nov.

Taxon classificationAnimaliaHymenopteraPteromalidae

AFEBC0F4-E62D-5837-8CC4-FB47A17E810A

http://zoobank.org/81FBA9DA-A6A7-4DF5-914C-64A27136D423

Fig. 5A–C

##### Material examined.

***Holotype*** Kenya • 1 ♀; Kakamega Forest, Kenya; 00°14'22.9N, 34°51'21E; 1594 m a.s.l.; 24 Jul. 2007; Hita-Garcia, F. leg.; Winkler extraction; Transect 12; ZFMK-HYM-00037130. ***Paratypes*** Kenya • 2 ♀; same data as for holotype; ZFMK-HYM-00037131 to ZFMK-HYM-00037132 • 1 ♀; Kakamega Forest, Kenya; 00°22'45N, 34°49'40.8E; 1618 m a.s.l.; 11 Sep. 2007; Hita-Garcia, F. leg.; Winkler extraction; Transect 27; ZFMK-HYM-00037133 • 1 ♀; Kakamega Forest, Kenya; 00°18'13.4N, 34°48'16E; 1554 m a.s.l.; 20 Jun. 2007; Hita-Garcia, F. leg.; Winkler extraction; Transect 5; ZFMK-HYM-00037134 • 1 ♀; Kakamega Forest, Kenya; 00°13'59.1N, 34°51'43.7E; 1614 m a.s.l.; 29 Aug. 2007; Hita-Garcia, F. leg.; Winkler extraction; Transect 24; ZFMK-HYM-00037135 • 1 ♀; same locality as for holotype; 17 Jul. 2007; Hita-Garcia, F. leg.; Winkler extraction; Transect 12; NHMUK013457232 • 1 ♀; Kakamega Forest, Kenya; 00°27'10.6N, 34°51'48.7E; 1676 m a.s.l.; 19 Jun. 2007; Hita-Garcia, F. leg.; Winkler extraction; Transect 4; NHMUK013457233 • 1 ♀; Kakamega Forest, Kenya; 00°36'9.7N, 34°37'20.3E; 1513 m a.s.l.; 07 Sep. 2007; Hita-Garcia, F. leg.; Winkler extraction; Transect 26; NMK: ZFMK-HYM-00037138 • 1 ♀; Kakamega Forest, Kenya; 00°33'17.9N, 34°40'55.9E; 1425 m a.s.l.; 20 Jun. 2008; Hita-Garcia, F. leg.; Winkler extraction; Transect 40; NMK: ZFMK-HYM-00037139.

**Female** (specimens used for morphometric measurements: ZFMK-HYM-00037130 to ZFMK-HYM-00037134).

##### Diagnosis.

**Figure 5.**
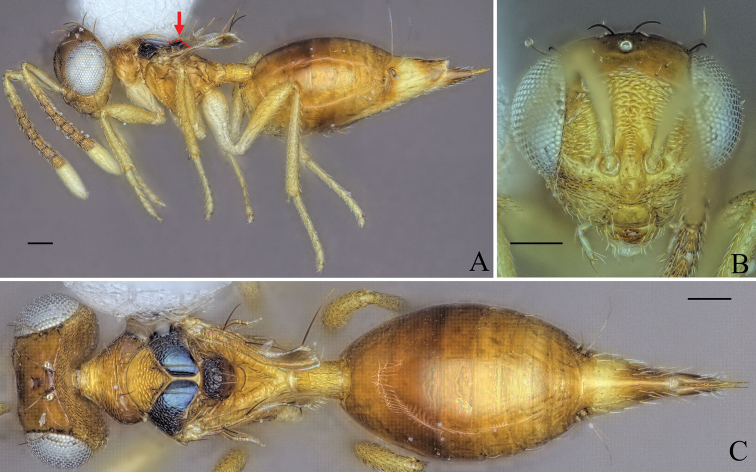
Holotype of *Diparaandreabalzerae* sp. nov. **A** habitus in lateral view **B** face in frontal view **C** body in dorsal view; red arrow: angle formed by anterior part of mesoscutellum and frenum. Scale bar: 100 µm.

Body brown to yellowish brown (Fig. 5); mesoscutellum black, raised, anterior part of mesoscutellum and frenum forming an angle of 120–125° (122°) (specimens used for measurement: ZFMK-HYM-00037130, ZFMK-HYM-00037132 to ZFMK-HYM-00037134) in lateral view (Fig. 5A); propodeum medially smooth and laterally transversely carinate (Fig. 5C).

##### Description.

***Size*:** small to medium sized, body length 1619–2183 (1809) µm.

***Coloration*:** body brown to yellowish brown (Fig. 5); dorsal part of scape, pedicel, first funicular segment, and clava yellowish white, ventral part of scape white, other funicular segments uniformly brown (Fig. 5A); vertex with bluish metallic tint (Fig. 5C); pronotum laterally dark brown (Fig. 5C); lateral area of mesoscutum black (Fig. 5C); mesoscutellum black with metallic tint, frenum dark brown (Fig. 5C); coxa, trochanter and proximal quarter of femur white, rest of legs pale yellowish brown (Fig. 5A); gts lighter from gt1 to gt6 (Fig. 5C); gt7 with pale yellowish brown coloration on anterior 1/2 and brown coloration on posterior 1/2 (Fig. 5C); ovipositor sheath brown (Fig. 5A).

***Head*:** head round, 1.26–1.31× (1.31) wider than high (Fig. 5B); vertex very sparsely foveolate and otherwise smooth (Fig. 5B); upper face strigate-reticulate (Fig. 5B); lower face reticulate, sparsely setose (Fig. 5B); distance of antennal insertion to eye short, 0.45–0.76× (0.45) (specimens used for measurement: ZFMK-HYM-00037130, ZFMK-HYM-00037132 to ZFMK-HYM-00037134) torulus diameter (Fig. 5B); antennae close, toruli separated by 0.84–1.07× (0.91) (specimens used for measurement: ZFMK-HYM-00037130, ZFMK-HYM-00037132 to ZFMK-HYM-00037134) torulus diameter (Fig. 5B); antennal formula: 11173 (Fig. 5A); funicle segments ~ as long as wide (Fig. 5A); malar space 0.29–0.35× (0.33) eye height (Fig. 5A); POL 0.62–0.74× (0.72) OOL (Fig. 5C).

***Mesosoma*:** pronotum large and elongated, 1.99–2.14× (2.02) as wide as long, substrigate, with a pair of setae close to the posterior edge (Fig. 5C); mesosoma slender, head breadth 1.53–1.59× (1.56) mesoscutum breadth (Fig. 5C); notauli converging ca. at 1/2 the length of mesoscutum (Fig. 5C); median area of mesoscutum reticulate (Fig. 5C); lateral area medially smooth and laterally reticulate (Fig. 5C); mesoscutum with two pairs of bristles, one pair of very large bristles on median area just anterior of notauli, almost reaching the mesoscutellum, and one pair laterally on lateral area anterior of wing base (Fig. 5C); axillae reticulate (Fig. 5C); mesoscutellum reticulate-rugulose, raised, with two pairs of bristles, one pair anterio-medially and one pair laterally just anterior of frenal line, anterior part of mesoscutellum and frenum forming an angle of 120–125° (122°) (specimens used for measurement: ZFMK-HYM-00037130, ZFMK-HYM-00037132 to ZFMK-HYM-00037134) (Fig. 5A); propodeum medially smooth and laterally transversely carinate (Fig. 5C); nucha with a few longitudinal carinae (Fig. 5C); brachypterous, fore wing reaching middle of petiole, tip truncated, with two or three large brown bristles and one large black bristle at the tip, with infuscation at tip (Fig. 5A).

***Metasoma*:** petiole short, 1.25–1.44× (1.29) longer than wide in dorsal view, costate-rugose, with lateral pair of large white setae visible in dorsal view (Fig. 5C); gaster medium, 1.23–1.56× (1.56) longer than mesosoma in dorsal view (Fig. 5C); gt1 covering ~ 1/3 of gaster, gt2–4 ca. equal in size, gt5 and 6 much smaller (Fig. 5C); gt7 and ovipositor sheath sparsely setose (Fig. 5A).

##### Remarks.

*Diparaandreabalzerae* is similar to *D.albomaculata*, *D.fastigata*, *D.nigroscutellata*, and *D.saetosa* in having a black mesoscutellum while the general body coloration is not black. *Diparaandreabalzerae* differs from *D.albomaculata*, *D.nigroscutellata* and *D.saetosa* in different propodeum sculpture. It differs from *D.fastigata* in body coloration, which is much darker in *D.fastigata* and the more obtuse angle formed by the anterior part of the mesoscutellum and the frenum in lateral view.

##### Male.

Unknown.

##### Etymology.

As the first author, I dedicate this species to my mother, Andrea Balzer, who sadly passed away in 2017.

##### Biology.

***Habitat*:** Leaf litter.

***Host*:** Unknown.

##### Distribution.

Kenya.

#### Dipara corona sp. nov.

Taxon classificationAnimaliaHymenopteraPteromalidae

A3F88B70-4E72-5CD2-B38F-BCC935099682

http://zoobank.org/4FDFD9C7-64C0-47EA-97A6-7587DA5E1BD4

Fig. 6A–C

##### Material examined.

***Holotype*** Kenya • 1 ♀; Kakamega Forest, Kenya; 00°14'52.3N, 34°52'5.3E; 1607 m a.s.l.; 21 Aug. 2007; Hita-Garcia, F. leg.; Winkler extraction; Transect 18; ZFMK-HYM-00040381.

##### Diagnosis.

**Figure 6.**
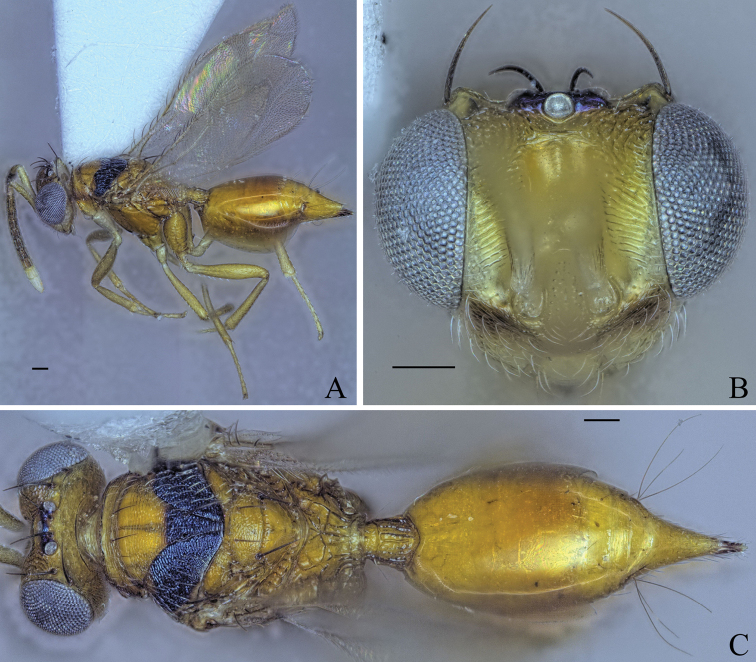
Holotype of *Diparacorona* sp. nov. **A** habitus in lateral view **B** face in frontal view **C** body in dorsal view. Scale bar: 100 µm.

**Female.** Broad dark brown stripe across head from one eye to the other below toruli (Fig. 6B); median and lateral area of mesoscutum with distinct transverse broad black stripe (Fig. 6C); macropterous, fore wing reaching gt7 (Fig. 6A); petiole 1.20× longer than wide in dorsal view (Fig. 6C).

##### Description.

***Size*:** medium sized, body length 2346 µm.

***Coloration*:** body brown to orangish brown (Fig. 6); scape and pedicel yellowish brown, funicle segments dark brown, clava white (Fig. 6); broad dark brown stripe across head from one eye to the other below toruli (Fig. 6B); vertex between ocelli black with metallic tint (Fig. 6B); procoxa white, rest of the legs yellowish brown (Fig. 6A); median and lateral area of mesoscutum with distinct transverse broad black stripe (Fig. 6C); tip of ovipositor sheath dark brown (Fig. 6A).

***Head*:** head oval, 1.33× wider than high (Fig. 6B); upper face substrigate (Fig. 6B); lower face substrigate around dark brown stripe and smooth below, sparsely setose (Fig. 6B); antennal scrobe and interantennal area smooth (Fig. 6B); distance of antennal insertion to eye short, 0.49× torulus diameter (Fig. 6B); antennae close, toruli separated by 1.31× torulus diameter (Fig. 6B); antennal formula: 11173 (Fig. 6A); funicle segments getting continuously shorter: f1 longer than wide to f7 as wide as long (Fig. 6A); malar space 0.33× eye height (Fig. 6A); vertex rugose, between ocelli slightly raised (Fig. 6B); occipital margin forming sharp edge (Fig. 6A); POL 1.31× OOL (Fig. 6C).

***Mesosoma*:** pronotum of medium length, 3.01× wider than long, substrigate, with a pair of bristles medially close to the posterior edge (Fig. 6C); mesosoma robust, head breadth 1.12× mesoscutum breadth (Fig. 6C); notauli not converging (Fig. 6C); mesoscutum with median area substrigate, lateral area strigate-reticulate, with two pairs of bristles: one pair on posterior 1/3 of medium area between notauli reaching axillae, one pair laterally on lateral area anterior of wing base (Fig. 6C); axillae reticulate (Fig. 6C); mesoscutellum reticulate, frenum smooth, with two pairs of bristles: one pair anteriorly and one pair laterally on frenal line (Fig. 6C); macropterous, fore wing reaching gt7, with large bristles along submarginal vein and smaller bristles along marginal and postmarginal vein on edge, transparent, stigmal vein long, stigma thin, uncus thin and pointed (Fig. 6A); propodeum with some transverse and longitudinal carinae (Fig. 6C); nucha carinate (Fig. 6C).

***Metasoma*:** petiole short, 1.20× longer than wide in dorsal view, anterior quarter constricted and rugose, rest costate, with lateral pair of large white setae visible in dorsal view (Fig. 6C); gaster short, 1.07× longer than mesosoma in dorsal view (Fig. 6C); gt1 covering ~ 2/3 of gaster (Fig. 6C); gt7 and ovipositor sheath sparsely setose (Fig. 6C).

##### Remarks.

*Diparacorona* is similar to *D.turneri* in having a distinct transverse broad black stripe on the median and lateral areas of the mesoscutum. In other not completely black species the black spots on the mesoscutum are restricted to the lateral area.

*Diparacorona* differs from *D.turneri* in the wing form and in the different petiole shape. The petiole is distinctly longer than wide in *D.corona* and slightly wider than long in *D.turneri*. Other differences include the body coloration, the shape of the mesoscutellum and the shape of the metacoxa.

##### Male.

Unknown.

##### Etymology.

Named after the Latin word *corona* for crown because of the raised and shiny part between the ocelli in frontal view, and additionally as a reference to the pandemic in 2020 and the following years caused by SARS-CoV-2, also known as the Corona virus.

##### Biology.

***Habitat*:** Leaf litter.

***Host*:** Unknown.

##### Distribution.

Kenya.

#### Dipara fastigata sp. nov.

Taxon classificationAnimaliaHymenopteraPteromalidae

1943FEB1-9D88-5B79-81A4-3D2C0012C5FF

http://zoobank.org/5AF8BF53-08A5-47DF-9B02-BADE13B8AC9E

Fig. 7A–C

##### Material examined.

***Holotype*** Kenya • 1 ♀; Kakamega Forest, Kenya; 00°14'6.1N, 34°52'9.2E; 1605 m a.s.l.; 28 Aug. 2007; Hita-Garcia, F. leg.; Winkler extraction; Transect 23; ZFMK-HYM-00040382.

##### Diagnosis.

**Figure 7.**
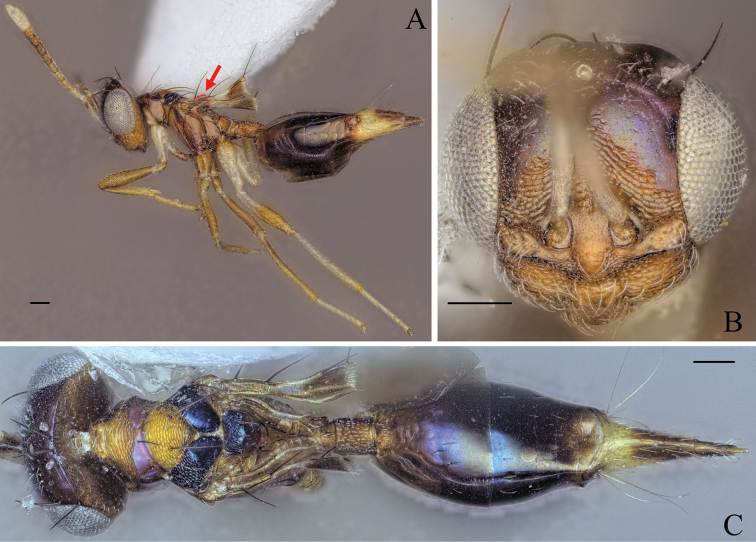
Holotype of *Diparafastigata* sp. nov. **A** habitus in lateral view **B** face in frontal view **C** body in dorsal view; red arrow: angle formed by anterior part of mesoscutellum and frenum. Scale bar: 100 µm.

**Female.** Body brown to dark brown (Fig. 7); mesoscutellum black, raised, anterior part of mesoscutellum and frenum forming an angle of 90° in lateral view (Fig. 7A); propodeum medially smooth and laterally transversely carinate (Fig. 7C).

##### Description.

***Size*:** small sized, body length 1946 µm.

***Coloration*:** body brown to dark brown (Fig. 7); upper face and vertex dark brown, lower face brown (Fig. 7B); scape dorsally dark brown and ventrally white, pedicel, f1, f2 and f3 yellowish brown, other funicle segments brown, clava white (Fig. 7A); fore leg with coxa white and rest yellowish brown (Fig. 7A); mid leg yellowish brown (Fig. 7A); hind leg with base of coxa, distal 1/2 of femur and distal tip of tibia yellowish brown, rest white (Fig. 7A); gt1–6 dark brown, posterior 1/2 of gt7 dorsally and ovipositor sheath brown, rest of gt7 pale yellowish white (Fig. 7A).

***Head*:** head round, 1.25× wider than high (Fig. 7B); vertex and upper 1/2 of upper face smooth, lower 1/2 laterally smooth and medially reticulate (Fig. 7B); lower face reticulate with smooth transverse stripe just below toruli from one eye to the other (Fig. 7B); upper 1/2 of upper face and lower face sparsely setose (Fig. 7B); antennal scrobe reticulate (Fig. 7B); interantennal area smooth (Fig. 7B); distance of antennal insertion to eye long, 1.12× torulus diameter (Fig. 7B); antennae far apart, toruli separated by 1.48× torulus diameter (Fig. 7B); antennal formula: 11173 (Fig. 7A); shape of funicle segments changing: f1 longer than wide to f7 ca. as wide as long (Fig. 7A); malar space 0.33× eye height (Fig. 7A); POL 0.85× OOL (Fig. 7C).

***Mesosoma*:** pronotum large and elongated, 1.93× wider than long, with a transverse carina, anteriorly of carina substrigate and posteriorly smooth, with a pair of setae posterio-medially (Fig. 7C); mesosoma slender, head breadth 1.64× mesoscutum breadth (Fig. 7C); notauli converging ca. at 2/3 of the length of mesoscutum (Fig. 7C); mesoscutum with median area reticulate, lateral area laterally reticulate and medially smooth, with two pairs of bristles: one pair on median area, reaching mesoscutellum, one pair on lateral area anterior of wing base (Fig. 7C); axillae reticulate (Fig. 7C); mesoscutellum raised, reticulate-rugulose, with one pair of bristles medially anterior of frenal line, anterior part of mesoscutellum and frenum forming an angle of 90° in lateral view (Fig. 7A); brachypterous, fore wing reaching middle of petiole, with 4 large bristles on the edge, upper and lower 1/3 infuscate, middle part transparent, tip truncated (Fig. 7A); propodeum medially smooth and laterally transversely carinate (Fig. 7C); nucha carinate (Fig. 7C).

***Metasoma*:** petiole medium, 1.6× longer than wide in dorsal view, reticulate, with two pairs of setae laterally visible in dorsal view (Fig. 7C); gaster medium, 1.31× longer than mesosoma in dorsal view (Fig. 7C); gt1 covering ~ 1/3 of gaster, gt2 much larger than following gts; gt3–5 ca. equal in size, gt6 much smaller (Fig. 7C); gt7 and ovipositor sheath sparsely setose (Fig. 7A).

##### Remarks.

*Diparafastigata* is similar to *D.andreabalzerae*, *D.albomaculata*, *D.nigroscutellata* and *D.saetosa* in having a black mesoscutellum while the general body coloration is not black. *Diparafastigata* differs from *D.albomaculata*, *D.nigroscutellata* and *D.saetosa* in different propodeum sculpture. It differs from *D.andreabalzerae* in general body coloration, which is much lighter in *D.andreabalzerae*, and the 90° angle formed by the anterior part of the mesoscutellum and the frenum in lateral view.

##### Male.

Unknown.

##### Etymology.

Named after the Latin adjective *fastigatus* for pointed or sharp. The name refers to the raised mesoscutellum.

##### Biology.

***Habitat*:** Leaf litter.

***Host*:** Unknown.

##### Distribution.

Kenya.

#### Dipara kakamegensis sp. nov.

Taxon classificationAnimaliaHymenopteraPteromalidae

5E7EBB67-C610-53B7-B1B5-5F6718F4A0A1

http://zoobank.org/72E6846C-B5E8-424F-BDC3-0D223E05CDC1

Fig. 8A–C

##### Material examined.

***Holotype*** Kenya • 1 ♀; Kakamega Forest, Kenya; 00°21'4.9N, 34°51'41.1E; 1602 m a.s.l.; Hita-Garcia, F. leg.; Winkler extraction; Transect 1; ZFMK-HYM-00037140. ***Paratypes*** Kenya • 4 ♀; Kakamega Forest, Kenya; 00°19'49.9N, 34°52'16.1E; 1580 m a.s.l.; 07 Aug. 2007; Hita-Garcia, F. leg.; Winkler extraction; Transect 15; ZFMK-HYM-00037141, ZFMK-HYM-00037198, ZFMK-HYM-00037199; NMK: ZFMK-HYM-00037200 • 4 ♀; Kakamega Forest, Kenya; 00°14'6.1N, 34°52'9.2E; 1605 m a.s.l.; 28 Aug. 2007; Hita-Garcia, F. leg.; Winkler extraction; Transect 23; ZFMK-HYM-00037142, ZFMK-HYM-00037241 to ZFMK-HYM-00037243 • 6 ♀; Kakamega Forest, Kenya; 00°21'4.4N, 34°51'41.1E; 1602 m a.s.l.; 07 Jun. 2007; Hita-Garcia, F. leg.; Winkler extraction; Transect 2; ZFMK-HYM-00037143, ZFMK-HYM-00037146, ZFMK-HYM-00037170, ZFMK-HYM-00037204 to ZFMK-HYM-00037206 • 1 ♀; Kakamega Forest, Kenya; 00°27'0.9N, 34°50'52.9E; 1649 m a.s.l.; 03 Jul. 2007; Hita-Garcia, F. leg.; Winkler extraction; Transect 8; ZFMK-HYM-00037144 • 1 ♀; Kakamega Forest, Kenya; 00°37'24.1N, 34°51'12E; 1585 m a.s.l.; 08 Aug. 2007; Hita-Garcia, F. leg.; Winkler extraction; Transect 10; ZFMK-HYM-00037145 • 8 ♀; Kakamega Forest, Kenya; 00°14'20.5N, 34°51'52.8E; 1634 m a.s.l.; 10 Aug. 2007; Hita-Garcia, F. leg.; Winkler extraction; Transect 17; ZFMK-HYM-00037147, ZFMK-HYM-00037156, ZFMK-HYM-00037158, ZFMK-HYM-00037159, ZFMK-HYM-00037229 to ZFMK-HYM-00037232 • 4 ♀; Kakamega Forest, Kenya; 00°21'7.9N, 34°52'2.6E; 1597 m a.s.l.; 09 Jul. 2007; Hita-Garcia, F. leg.; Winkler extraction; Transect 7; ZFMK-HYM-00037148, ZFMK-HYM-00037151, ZFMK-HYM-00037154, ZFMK-HYM-00037193 • 7 ♀; Kakamega Forest, Kenya; 00°14'20.5N, 34°51'52.8E; 1634 m a.s.l.; 04 Aug. 2007; Hita-Garcia, F. leg.; Winkler extraction; Transect 17; ZFMK-HYM-00037149, ZFMK-HYM-00037164, ZFMK-HYM-00037233 to ZFMK-HYM-00037237 • 7 ♀; same data as for holotype; ZFMK-HYM-00037150, ZFMK-HYM-00037212 to ZFMK-HYM-00037217 • 9 ♀; Kakamega Forest, Kenya; 00°14'52.3N, 34°52'5.3E; 1607 m a.s.l.; 14 Aug. 2007; Hita-Garcia, F. leg.; Winkler extraction; Transect 18; ZFMK-HYM-00037152, ZFMK-HYM-00037157, ZFMK-HYM-00037162, ZFMK-HYM-00037165, ZFMK-HYM-00037173 to ZFMK-HYM-00037175; NHMUK013457217, NHMUK013457218 • 1 ♀; Kakamega Forest, Kenya; 00°19'36N, 34°52'14.6E; 1570 m a.s.l.; 21 Jun. 2007; Hita-Garcia, F. leg.; Winkler extraction; Transect 6; ZFMK-HYM-00037153 • 7 ♀; Kakamega Forest, Kenya; 00°21'7.9N, 34°52'2.6E; 1597 m a.s.l.; 02 Jul. 2007; Hita-Garcia, F. leg.; Winkler extraction; Transect 7; ZFMK-HYM-00037155, ZFMK-HYM-00037167, ZFMK-HYM-00037223 to ZFMK-HYM-00037227 • 2 ♀; Kakamega Forest, Kenya; 00°19'45.7N, 34°52'2.8E; 1573 m a.s.l.; 24 Aug. 2007; Hita-Garcia, F. leg.; Winkler extraction; Transect 21; ZFMK-HYM-00037160, ZFMK-HYM-00037244 • 4 ♀; Kakamega Forest, Kenya; 00°20'52.5N, 34°51'53E; 1592 m a.s.l.; 06 Sep. 2007; Hita-Garcia, F. leg.; Winkler extraction; Transect 25; ZFMK-HYM-00037161, ZFMK-HYM-00037218 to ZFMK-HYM-00037220 • 5 ♀; Kakamega Forest, Kenya; 00°19'49.9N, 34°52'16.1E; 1580 m a.s.l.; 01 Aug. 2007; Hita-Garcia, F. leg.; Winkler extraction; Transect 15; ZFMK-HYM-00037163, ZFMK-HYM-00037166; NHMUK013457219 to NHMUK013457221 • 4 ♀; Kakamega Forest, Kenya; 00°13'15.5N, 34°53'24.7E; 1597 m a.s.l.; 23 Aug. 2007; Hita-Garcia, F. leg.; Winkler extraction; Transect 22; ZFMK-HYM-00037168, ZFMK-HYM-00037188, ZFMK-HYM-00037190; NMK: ZFMK-HYM-00037189 • 1 ♀; Kakamega Forest, Kenya; 00°19'45.7N, 34°52'2.8E; 1573 m a.s.l.; 17 Aug. 2007; Hita-Garcia, F. leg.; Winkler extraction; Transect 21; ZFMK-HYM-00037169 • 3 ♀; Kakamega Forest, Kenya; 00°14'6.1N, 34°52'9.2E; 1605 m a.s.l.; 04 Sep. 2007; Hita-Garcia, F. leg.; Winkler extraction; Transect 23; ZFMK-HYM-00037179 to ZFMK-HYM-00037181 • 4 ♀; Kakamega Forest, Kenya; 00°14'52.3N, 34°52'5.3E; 1607 m a.s.l.; 21 Aug. 2007; Hita-Garcia, F. leg.; Winkler extraction; Transect 18; ZFMK-HYM-00037182 to ZFMK-HYM-00037185 • 1 ♀; Kakamega Forest, Kenya; 00°19'45.7N, 34°52'2.8E; 1573 m a.s.l.; 07 Aug. 2007; Hita-Garcia, F. leg.; Winkler extraction; Transect 21; ZFMK-HYM-00037186 • 1 ♀; Kakamega Forest, Kenya; 00°22'50.5N, 34°49'21.4E; 1623 m a.s.l.; 22 Aug. 2007; Hita-Garcia, F. leg.; Winkler extraction; Transect 19; ZFMK-HYM-00037187 • 4 ♀; Kakamega Forest, Kenya; 00°19'36N, 34°52'14.6E; 1570 m a.s.l.; 28 Jun. 2007; Hita-Garcia, F. leg.; Winkler extraction; Transect 6; ZFMK-HYM-00037194, ZFMK-HYM-00037195; NMK: ZFMK-HYM-00037196, ZFMK-HYM-00037197 • 3 ♀; Kakamega Forest, Kenya; 00°12'42.6N, 34°55'52.3E; 1615 m a.s.l.; 16 Aug. 2007; Hita-Garcia, F. leg.; Winkler extraction; Transect 20; ZFMK-HYM-00037202, ZFMK-HYM-00037203; NMK: ZFMK-HYM-00037201 • 5 ♀; Kakamega Forest, Kenya; 00°21'4.4N, 34°51'41.1E; 1602 m a.s.l.; 05 Jun. 2007; Hita-Garcia, F. leg.; Winkler extraction; Transect 2; ZFMK-HYM-00037207 to ZFMK-HYM-00037211 • 1 ♀; Kakamega Forest, Kenya; 00°14'22.9N, 34°51'21E; 1594 m a.s.l.; 17 Jul. 2007; Hita-Garcia, F. leg.; Winkler extraction; Transect 12; ZFMK-HYM-00037221 • 1 ♀; Kakamega Forest, Kenya; 00°37'24.1N, 34°51'12E; 1585 m a.s.l.; 01 Aug. 2007; Hita-Garcia, F. leg.; Winkler extraction; Transect 10; ZFMK-HYM-00037228 • 3 ♀; Kakamega Forest, Kenya; 00°21'21.1N, 34°51'44.9E; 1632 m a.s.l.; 08 Aug. 2007; Hita-Garcia, F. leg.; Winkler extraction; Transect 16; ZFMK-HYM-00037238 to ZFMK-HYM-00037240 • 3 ♀; Kakamega Forest, Kenya; 00°20'52.5N, 34°51'53E; 1592 m a.s.l.; 13 Sep. 2007; Hita-Garcia, F. leg.; Winkler extraction; Transect 25; ZFMK-HYM-00037245 to ZFMK-HYM-00037247.

**Female** (specimens used for morphometric measurements: ZFMK-HYM-00037140 to ZFMK-HYM-00037144).

##### Diagnosis.

**Figure 8.**
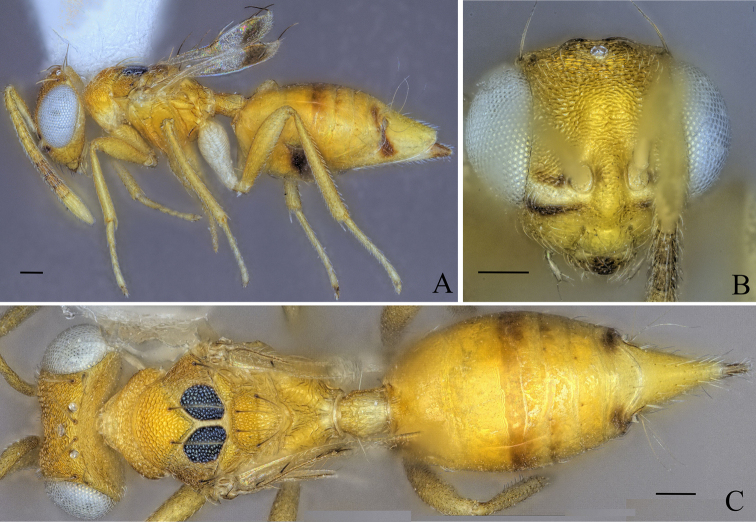
Holotype of *Diparakakamegensis* sp. nov. **A** habitus in lateral view **B** face in frontal view **C** body in dorsal view. Scale bar: 100 µm.

Body bright yellowish brown (Fig. 8); face with two transverse stripes of very dark brown coloration just at the level of toruli and at the level of the ventral margin of the eye, interrupted in interantennal area and supraclypeal area, enclosing a stripe of pale white coloration (Fig. 8B); legs yellowish brown except for metacoxa white (Fig. 8A); brachypterous, fore wing reaching middle of gt1 (Fig. 8A); mesoscutellum small, mesosoma length 3.90–4.86× (4.86) (Fig. 8C) (specimens used for measurement: ZFMK-HYM-00037140 to ZFMK-HYM-00037170) mesoscutellum length; petiole short to medium, 1.15–1.72× (1.15) as long as wide in dorsal view.

##### Description.

***Size*:** small to medium sized, body length 1483–2227 (2027) µm.

***Coloration*:** body bright yellowish brown (Fig. 8); ventral part of scape and clava pale yellowish white, dorsal part of scape and last three funicle segments brown, rest of funicle segments and pedicel yellowish brown (Fig. 8A); face with two transverse dark brown stripes just at the level of toruli and at the level of the ventral margin of the eye, interrupted in interantennal area and supraclypeal area, enclosing a stripe of pale white coloration (Fig. 8B); two black spots medially on lateral areas of mesoscutum (Fig. 8C); middle part and tip of the fore wing infuscate (Fig. 8A); legs yellowish brown except for metacoxa white (Fig. 8A); nucha and posterior 2/3 of petiole pale yellowish white, rest of petiole bright yellowish brown (Fig. 8C); some darker brown stripes dorsally on gaster (Fig. 8A); brown spots on gt6 and gt7 around cerci (Fig. 8A); tip of ovipositor sheath brown (Fig. 8A).

***Head*:** head round to oval, 1.25–1.63× (1.63) wider than high (Fig. 8B); vertex, upper face and interantennal area reticulate, antennal scrobe subreticulate, lower face smooth and sparsely setose (Fig. 8B); distance of antennal insertion to eye short, 0.78–0.97× (0.78) torulus diameter (Fig. 8B); antennae close, toruli separated by 1.15–1.31× (1.31) torulus diameter (Fig. 8B); antennal formula: 11173 (Fig. 8A); funicle segments slightly longer than wide (Fig. 8A); malar space 0.30–0.37× (0.30) eye height; occipital carina forming a sharp edge (Fig. 8A); POL 1.21–1.41× (1.38) OOL (Fig. 8C).

***Mesosoma*:** pronotum of medium length, 2.85–3.37× (3.37) as wide as long (Fig. 8C); mesosoma of medium breadth, head breadth 1.24–1.45× (1.36) mesoscutum breadth (Fig. 8C); pronotum, mesoscutum, axillae and mesoscutellum reticulate (Fig. 8C); notauli converging ca. at 2/3 of the length of mesoscutum (Fig. 8C); mesoscutum with two pairs of bristles: one pair of very large bristles on median area just anterior of notauli, reaching posterior edge of mesoscutum and one pair laterally on lateral area anterior of wing base (Fig. 8C); mesoscutellum small, mesosoma length 3.90–4.86× (4.86) (specimens used for measurement: ZFMK-HYM-00037140 to ZFMK-HYM-00037170) mesoscutellum length, with two pairs of bristles: one pair medially close to anterior edge of mesoscutellum and one pair laterally on the frenal line (Fig. 8C); propodeum medially rugose and laterally transversely carinate-rugose, extending to nucha (Fig. 8C); brachypterous, fore wing reaching middle of gt1, with five large black bristles along the edge and one to ten bristles on the tip (Fig. 8A) (holotype: seven).

***Metasoma*:** petiole short to medium, 1.15–1.72× (1.15) as long as wide in dorsal view, reticulate-rugose, with lateral pair of large white setae visible in dorsal view (Fig. 8C); gaster medium, 1.21–1.47× (1.47) longer than mesosoma in dorsal view (Fig. 8C); gt1 covering ~ 1/3 of gaster, gt2–4 ca. equal in size, gt5 and 6 much smaller (Fig. 8C); gt7 and ovipositor sheath sparsely setose (Fig. 8A).

##### Variations.

The bristles on the forewing can vary from five to 15. This variation is found in the bristles at the tip of the wing while along the edges there are constantly five bristles. In some specimens there are just a few larger bristles at the tip and in others there can be up to ten small bristles at the tip. The number of bristles can vary between left and right wing in one specimen. The surface sculpture of the median part of the propodeum can vary from rugose to smooth.

##### Remarks.

*Diparakakamegensis* is very similar to *D.nyani*. It differs from *D.nyani* in the following characters: *D.kakamegensis* is brachypterous and the mesoscutellum is smaller relative to the mesosoma length, based on the morphometric analysis (Fig. 3). The stripes across the face are similar in *D.maculata*, *D.reticulata* and *D.rodneymulleni*. *Diparakakamegensis* differs from *D.maculata* in having a yellowish brown mesocoxa and petiole. *Diparakakamegensis* differs from *D.rodneymulleni* in many characters: *D.kakamegensis* is brachypterous, the body coloration, the length of the petiole and general body shape. *Diparakakamegensis* differs from *D.reticulata* in having smooth gastral tergites while they are reticulated in *D.reticulata*.

##### Male.

Unknown.

##### Etymology.

Named after the collecting locality.

##### Biology.

***Habitat*:** Leaf litter.

***Host*:** Unknown.

##### Distribution.

Kenya.

#### Dipara lux sp. nov.

Taxon classificationAnimaliaHymenopteraPteromalidae

CF845FC5-274D-5D61-A098-5ECEA4B8F401

http://zoobank.org/0B638A88-80E4-4462-A215-9EE691A78A37

Fig. 9A–C

##### Material examined.

***Holotype*** Kenya • 1 ♀; Kakamega Forest, Kenya; 00°18'13.4N, 34°48'16E; 1554 m a.s.l.; 20 Jun. 2007; Hita-Garcia, F. leg.; Winkler extraction; Transect 5; ZFMK-HYM-00040379. ***Paratype*** Kenya • 1 ♀; same data as for holotype; ZFMK-HYM-00040380.

##### Diagnosis.

**Figure 9.**
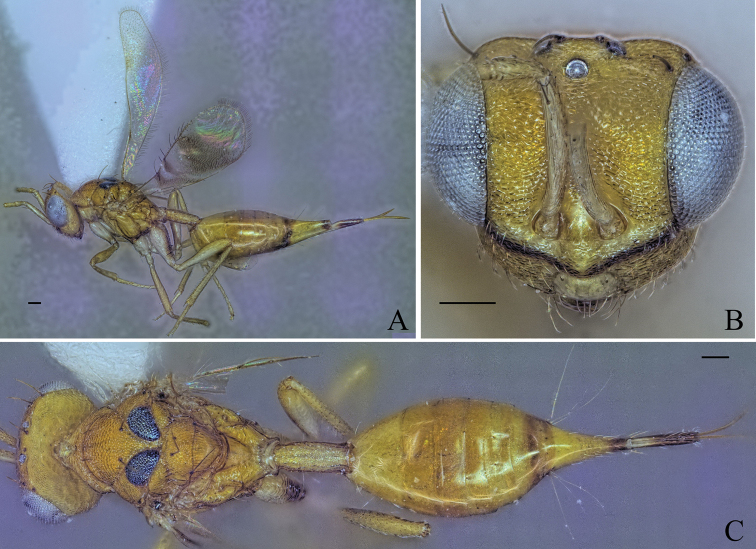
Holotype of *Diparalux* sp. nov. **A** habitus in lateral view **B** face in frontal view **C** body in dorsal view. Scale bar: 100 µm.

**Female.** Body yellowish brown (Fig. 9); face with dark brown to black stripe from one eye to the other at the level of the ventral margin of the eye (Fig. 9B); vertex reticulate (Fig. 9B); petiole very long, 2.50–2.61× (2.50) longer than wide in dorsal view (Fig. 9C).

##### Description.

***Size*:** medium sized, body length 2243–2772 (2772) µm.

***Coloration*:** body yellowish brown (Fig. 9); scape, pedicel and f1–3 yellowish brown, f4 yellowish brown to dark brown, f5–7 dark brown, clava yellowish brown (Fig. 9A); face with dark brown to black stripe from one eye to the other at the level of the ventral margin of the eye (Fig. 9B); mesoscutum with two black spots medially on lateral area (Fig. 9C); fore leg with distal tip of coxa brown and rest of coxa white, trochanter brown, rest yellowish brown (Fig. 9A); mid leg with coxa and trochanter white, rest yellowish brown (Fig. 9A); hind leg with anterior part of coxa white and posterior part dark brown, anterior part of femur white, rest yellowish brown (Fig. 9A); gt6 and gt7 with dark brown spots around cerci (Fig. 9A); posterior tip of gt7 dark brown (Fig. 9A); tip of ovipositor sheath dark brown, rest white (Fig. 9A).

***Head*:** head oval, 1.31–1.34× (1.34) wider than high, reticulate except for interantennal area smooth (Fig. 9B); lower face sparsely setose (Fig. 9B); distance of antennal insertion to eye short, 0.55–0.60× (0.55) torulus diameter (Fig. 9B); antennae close, toruli separated by 1.13–1.24× (1.13) torulus diameter (Fig. 9B); funicle segments slightly longer than wide (Fig. 9A); malar space 0.34–0.38× (0.34) eye height (Fig. 9A); POL 0.92–1.00× (1.00) OOL (Fig. 9A).

***Mesosoma*:** pronotum of medium length, 3.28–3.37× (3.37) as wide as long, reticulate, with two pairs of setae close to posterior edge (Fig. 9C); mesosoma robust, head breadth 1.16–1.19× (1.16) mesoscutum breadth (Fig. 9C); notauli converging at posterior margin of mesoscutum (Fig. 9C); mesoscutum reticulate, with two pairs of bristles: one pair on median area anterior of notauli, one pair laterally on lateral area anterior of wing base (Fig. 9C); axillae reticulate (Fig. 9C); mesoscutellum anteriorly reticulate, frenum carinate, with two pairs of bristles: one pair anterio-medially and one pair laterally anterior of frenal line (Fig. 9C); macropterous, fore wing with large bristles along marginal and postmarginal vein on edge, with dense brush of setae at proximal end of marginal vein, with large area of infuscation on distal part and smaller areas of infuscation medially, stigmal vein long, stigma small and rounded, uncus short (Fig. 9A); propodeum medially smooth and laterally transversely confused carinate (Fig. 9C); nucha carinate (Fig. 9C).

***Metasoma*:** petiole very long, 2.50–2.61× (2.50) longer than wide in dorsal view, areolate-rugose, with lateral pair of large white setae visible in dorsal view (Fig. 9C); gaster medium, 1.34–1.37× (1.34) longer than mesosoma in dorsal view; gt1 covering ~ 1/3 of gaster, gt2–4 ca. equal in size, gt5–6 smaller (Fig. 9C); gt7 and ovipositor sheath sparsely setose and elongated, together ~ 1/2 as long as rest of gaster (Fig. 9C).

##### Remarks.

*Diparalux* is similar to *D.corona*, *D.machadoi*, *D.striata*, *D.tenebra*, *D.tigrina* and *D.turneri* in having one dark brown to black stripe across the face. *Diparalux* is different from *D.machadoi* in having distinct notauli, which are lacking in *D.machadoi*. It differs from *D.corona*, *D.striata*, *D.turneri* and *D.tigrina* in having a very long petiole. *Diparalux* and *D.tenebra* are very similar in body shape and differ in their body coloration which is much brighter in *D.lux* and in the surface sculpture of the head. They share the otherwise unique character of having a dense brush of setae close to the proximal end of the marginal vein on the fore wing.

##### Male.

Unknown.

##### Etymology.

Named after the Latin word *lux* for light, in contrast to *D.tenebra* which looks very similar but has a darker coloration.

##### Biology.

***Habitat*:** Leaf litter.

***Host*:** Unknown.

##### Distribution.

Kenya.

#### Dipara nigroscutellata sp. nov.

Taxon classificationAnimaliaHymenopteraPteromalidae

34F5844A-3F58-5DA8-AE77-35B81144193A

http://zoobank.org/8CEE0099-F1CB-4CC4-B9FC-DFC523A6B639

Fig. 10A–C

##### Material examined.

***Holotype*** Kenya • 1 ♀; Kakamega Forest, Kenya; 00°19'49.9N, 34°52'16.1E; 1580 m a.s.l.; 07 Aug. 2007; Hita-Garcia, F. leg.; Winkler extraction; Transect 15; ZFMK-HYM-00037253. ***Paratypes*** Kenya • 5 ♀; Kakamega Forest, Kenya; 00°21'21.1N, 34°51'44.9E; 1632 m a.s.l.; 01 Aug. 2007; Hita-Garcia, F. leg.; Winkler extraction; Transect 16; ZFMK-HYM-00037254; NMK: ZFMK-HYM-00040266 to ZFMK-HYM-00040269 • 4 ♀; Kakamega Forest, Kenya; 00°20'52.5N, 34°51'53E; 1592 m a.s.l.; 06 Sep. 2007; Hita-Garcia, F. leg.; Winkler extraction; Transect 25; ZFMK-HYM-00037255, ZFMK-HYM-00040257, ZFMK-HYM-00040279, ZFMK-HYM-00040280 • 2 ♀; Kakamega Forest, Kenya; 00°27'10.6N, 34°51'48.7E; 1676 m a.s.l.; 19 Jun. 2007; Hita-Garcia, F. leg.; Winkler extraction; Transect 4; ZFMK-HYM-00037256, ZFMK-HYM-00040309 • 1 ♀; Kakamega Forest, Kenya; 00°21'21.1N, 34°51'44.9E; 1632 m a.s.l.; 08 Aug. 2007; Hita-Garcia, F. leg.; Winkler extraction; Transect 16; NHMUK013457222 • 1 ♀; Kakamega Forest, Kenya; 00°13'15.5N, 34°53'24.7E; 1597 m a.s.l.; 25 Aug. 2007; Hita-Garcia, F. leg.; Winkler extraction; Transect 22; NHMUK013457223 • 1 ♀; Kakamega Forest, Kenya; 00°14'52.3N, 34°52'5.3E; 1607 m a.s.l.; 14 Aug. 2007; Hita-Garcia, F. leg.; Winkler extraction; Transect 18; NHMUK013457224 • 1 ♀; Kakamega Forest, Kenya; 00°13'15.5N, 34°53'24.7E; 1597 m a.s.l.; 23 Aug. 2007; Hita-Garcia, F. leg.; Winkler extraction; Transect 22; NHMUK013457225 • 2 ♀; Kakamega Forest, Kenya; 00°19'45.7N, 34°52'2.8E; 1573 m a.s.l.; 24 Aug. 2007; Hita-Garcia, F. leg.; Winkler extraction; Transect 21; NHMUK013457226; ZFMK-HYM-00040263 • 1 ♀; Kakamega Forest, Kenya; 00°23'6.2N, 34°33'37.8E; 1602 m a.s.l.; 16 Jul. 2007; Hita-Garcia, F. leg.; Winkler extraction; Transect 11; ZFMK-HYM-00040264 • 1 ♀; Kakamega Forest, Kenya; 00°14'20.5N, 34°51'52.8E; 1634 m a.s.l.; 04 Aug. 2007; Hita-Garcia, F. leg.; Winkler extraction; Transect 17; ZFMK-HYM-00040265 • 1 ♀; Kakamega Forest, Kenya; 00°12'42.6N, 34°55'52.3E; 1615 m a.s.l.; 10 Aug. 2007; Hita-Garcia, F. leg.; Winkler extraction; Transect 20; NMK: ZFMK-HYM-00040270 • 9 ♀; Kakamega Forest, Kenya; 00°21'4.4N, 34°51'41.1E; 1602 m a.s.l.; 05 Jun. 2007; Hita-Garcia, F. leg.; Winkler extraction; Transect 2; ZFMK-HYM-00040271 to ZFMK-HYM-00040275, ZFMK-HYM-00040301 to ZFMK-HYM-00040304 • 1 ♀; Kakamega Forest, Kenya; 00°21'4.9N, 34°51'41.1E; 1602 m a.s.l.; Hita-Garcia, F. leg.; Winkler extraction; Transect 1; ZFMK-HYM-00040276 • 2 ♀; Kakamega Forest, Kenya; 00°14'6.1N, 34°52'9.2E; 1605 m a.s.l.; 04 Sep. 2007; Hita-Garcia, F. leg.; Winkler extraction; Transect 23; ZFMK-HYM-00040277, ZFMK-HYM-00040278 • 1 ♀; Kakamega Forest, Kenya; 00°27'0.9N, 34°50'52.9E; 1649 m a.s.l.; 10 Jul. 2007; Hita-Garcia, F. leg.; Winkler extraction; Transect 8; ZFMK-HYM-00040281 • 3 ♀; Kakamega Forest, Kenya; 00°21'7.9N, 34°52'2.6E; 1597 m a.s.l.; 02 Jul. 2007; Hita-Garcia, F. leg.; Winkler extraction; Transect 7; ZFMK-HYM-00040282, ZFMK-HYM-00040299, ZFMK-HYM-00040300 • 1 ♀; Kakamega Forest, Kenya; 00°14'52.3N, 34°52'5.3E; 1607 m a.s.l.; 21 Aug. 2007; Hita-Garcia, F. leg.; Winkler extraction; Transect 18; ZFMK-HYM-00040283 • 1 ♀; Kakamega Forest, Kenya; 00°19'45.7N, 34°52'2.8E; 1573 m a.s.l.; 07 Aug. 2007; Hita-Garcia, F. leg.; Winkler extraction; Transect 21; ZFMK-HYM-00040284 • 4 ♀; Kakamega Forest, Kenya; 00°21'7.9N, 34°52'2.6E; 1597 m a.s.l.; 09 Jul. 2007; Hita-Garcia, F. leg.; Winkler extraction; Transect 7; ZFMK-HYM-00040285 to ZFMK-HYM-00040288 • 10 ♀; Kakamega Forest, Kenya; 00°21'4.4N, 34°51'41.1E; 1602 m a.s.l.; 07 Jun. 2007; Hita-Garcia, F. leg.; Winkler extraction; Transect 2; ZFMK-HYM-00040289 to ZFMK-HYM-00040298 • 2 ♀; Kakamega Forest, Kenya; 00°14'20.5N, 34°51'52.8E; 1634 m a.s.l.; 10 Aug. 2007; Hita-Garcia, F. leg.; Winkler extraction; Transect 17; ZFMK-HYM-00040305, ZFMK-HYM-00040306 • 1 ♀; Kakamega Forest, Kenya; 00°22'45N, 34°49'40.8E; 1618 m a.s.l.; 11 Sep. 2007; Hita-Garcia, F. leg.; Winkler extraction; Transect 27; ZFMK-HYM-00040307 • 1 ♀; Kakamega Forest, Kenya; 00°19'36N, 34°52'14.6E; 1570 m a.s.l.; 21 Jun. 2007; Hita-Garcia, F. leg.; Winkler extraction; Transect 6; ZFMK-HYM-00040308 • 15 ♀; Kakamega Forest, Kenya; 00°19'49.9N, 34°52'16.1E; 1580 m a.s.l.; 07 Aug. 2007; Hita-Garcia, F. leg.; Winkler extraction; Transect 15; ZFMK-HYM-00040310 to ZFMK-HYM-00040324 • 14 ♀; Kakamega Forest, Kenya; 00°19'49.9N, 34°52'16.1E; 1580 m a.s.l.; 01 Aug. 2007; Hita-Garcia, F. leg.; Winkler extraction; Transect 15; ZFMK-HYM-00040325 to ZFMK-HYM-00040338.

**Female** (specimens used for morphometric measurements: ZFMK-HYM-00037253 to ZFMK-HYM-00037256, ZFMK-HYM-00040257).

##### Diagnosis.

**Figure 10.**
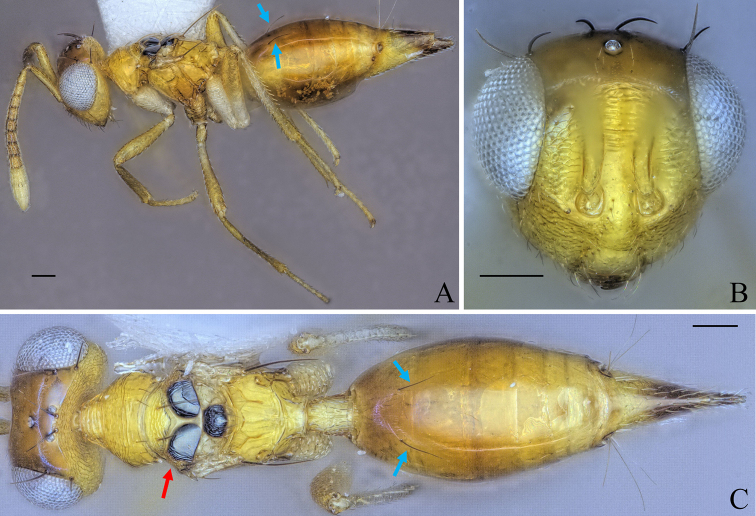
Holotype of *Diparanigroscutellata* sp. nov. **A** habitus in lateral view **B** face in frontal view **C** body in dorsal view; red arrow: yellowish brown area on lateral area of mesoscutum; blue arrows: bristles on gt1. Scale bar: 100 µm.

Body yellowish brown to brown (Fig. 10); lateral area of mesoscutum almost completely black, small area laterally yellowish brown (Fig. 10C); mesoscutellum black (Fig. 10C); gt1 with a pair of large bristles dorso-anteriorly (Fig. 10A and C).

##### Description.

***Size*:** small sized, body length 1653–2015 (1815) µm.

***Coloration*:** body yellowish brown to brown (Fig. 10); face yellowish brown and vertex brown (Fig. 10B); scape, pedicel and f1 yellowish brown, f2–7 brown, clava pale yellowish brown (Fig. 10A); lateral area of mesoscutum almost completely black, small area laterally yellowish brown (Fig. 10C); axillae white (Fig. 10C); fore leg with coxa white and rest yellowish brown (Fig. 10A); mid leg with proximal 1/3 of femur white, distal 1/2 of tibia brown, rest yellowish brown (Fig. 10A); hind leg with coxa, proximal 1/2 of femur and proximal 1/2 of tibia white, rest yellowish brown (Fig. 10A); color gradient on gaster, from brown (gt1) to yellowish brown (gt7) (Fig. 10C); posterior 1/2 of gt7 and tip of ovipositor sheath dark brown, rest of gt7 yellowish brown (Fig. 10A).

***Head*:** head round, 1.19–1.26× (1.21) wider than high (Fig. 10B); upper and lower face reticulate, lower face sparsely setose (Fig. 10B); vertex and interantennal area smooth (Fig. 10B); antennal scrobe strigate-reticulate (Fig. 10B); insertion point of antenna same level as ventral margin of eye (Fig. 10B); antennae close, toruli separated by 1.03–1.19× (1.04) torulus diameter (Fig. 10B); antennal formula: 11173 (Fig. 10A); funicle segments ca. as long as wide (Fig. 10A); malar space 0.37–0.40× (0.40) eye height (Fig. 10A); POL 0.60–0.94× (0.60) OOL (Fig. 10C).

***Mesosoma*:** pronotum large and elongated, 1.48–1.65× (1.48) as wide as long, strigate, with two or three pairs of setae laterally close to the posterior edge (Fig. 10C); mesosoma slender, head breadth 1.64–1.71× (1.65) mesoscutum breadth (Fig. 10C); notauli converging ca. at 1/2 the length of mesoscutum (Fig. 10C); median area of mesoscutum strigate-reticulate, black spots on lateral area mostly smooth except for lateral edges carinate-reticulate, lateral area laterally reticulate (Fig. 10C); mesoscutum with two pairs of bristles: one pair on median area, reaching posterior edge of mesoscutum, one pair laterally on lateral area anterior of wing base (Fig. 10C); axillae mostly smooth with some confused ridges (Fig. 10C); mesoscutellum and black spots on lateral area slightly raised (Fig. 10A); mesoscutellum reticulate-rugulose with two pairs of bristles: one pair medially and one small pair posterio-laterally (Fig. 10C); brachypterous, fore wing very small, reaching propodeum, with a large black bristle at the tip (Fig. 10A); propodeum completely smooth (Fig. 10C); nucha smooth with a few longitudinal carinae (Fig. 10C).

***Metasoma*:** petiole short, 0.98–1.16× (1.06) as long as wide, costate-rugose, with lateral pair of large white setae visible in dorsal view (Fig. 10C); gt1 with a pair of large bristles dorso-anteriorly (Fig. 10A and C); gaster medium, 1.53–1.75× (1.59) longer than mesosoma in dorsal view (Fig. 10C); gt1 covering ~ 1/3 of gaster, gts smaller from gt2 to gt6 (Fig. 10C); gt7 and ovipositor sheath sparsely setose (Fig. 10A).

##### Variation.

The bristles on the gt1 and the tip of the forewing can sometimes be missing. In this case the pit where the bristles are supposed to be is still visible.

##### Remarks.

*Diparanigroscutellata* is similar to *D.andreabalzerae*, *D.albomaculata*, *D.fastigata*, and *D.saetosa* in having a black mesoscutellum while the general body coloration is not black. *Diparanigroscutellata* differs from *D.andreabalzerae* and *D.fastigata* in having a pair of large bristles dorso-anteriorly on the gt1. It differs from *D.albomaculata* and *D.saetosa* in the general body coloration, which is much brighter in *D.nigroscutellata* and in the coloration of the lateral area of the mesoscutum. In *D.nigroscutellata* the lateral area is laterally yellowish brown and in *D.albomaculata* and *D.saetosa* the lateral area is completely black.

*Diparanigroscutellata* is similar to *D.straminea* in sharing the bristles on the gt1 and in propodeum sculpture. It differs from *D.straminea* in having a black mesoscutellum.

##### Male.

Unknown.

##### Etymology.

Named after the black mesoscutellum.

##### Biology.

***Habitat*:** Leaf litter.

***Host*:** Unknown.

##### Distribution.

Kenya.

#### Dipara nyani sp. nov.

Taxon classificationAnimaliaHymenopteraPteromalidae

44D60DB9-2F0F-55C4-AAE6-2B812ABC5ADE

http://zoobank.org/3290D412-E90C-4638-872F-F6CD6D8290CA

Fig. 11A–C

##### Material examined.

***Holotype*** Kenya • 1 ♀; Kakamega Forest, Kenya; 00°19'45.7N, 34°52'2.8E; 1573 m a.s.l.; 17 Aug. 2007; Hita-Garcia, F. leg.; Winkler extraction; Transect 21; ZFMK-HYM-00037248. ***Paratypes*** Kenya • 1 ♀; Kakamega Forest, Kenya; 00°21'21.1N, 34°51'44.9E; 1632 m a.s.l.; 01 Aug. 2007; Hita-Garcia, F. leg.; Winkler extraction; Transect 16; ZFMK-HYM-00037249 • 1 ♀; Kakamega Forest, Kenya; 00°19'45.7N, 34°52'2.8E; 1573 m a.s.l.; 24 Aug. 2007; Hita-Garcia, F. leg.; Winkler extraction; Transect 21; NHMUK013457234 • 1 ♀; Kakamega Forest, Kenya; 00°19'45.7N, 34°52'2.8E; 1573 m a.s.l.; 17 Aug. 2007; Hita-Garcia, F. leg.; Winkler extraction; Transect 21; NMK: ZFMK-HYM-00037251 • 1 ♀; Kakamega Forest, Kenya; 00°13'15.5N, 34°53'24.7E; 1597 m a.s.l.; 23 Aug. 2007; Hita-Garcia, F. leg.; Winkler extraction; Transect 22; ZFMK-HYM-00037252.

##### Diagnosis.

**Figure 11.**
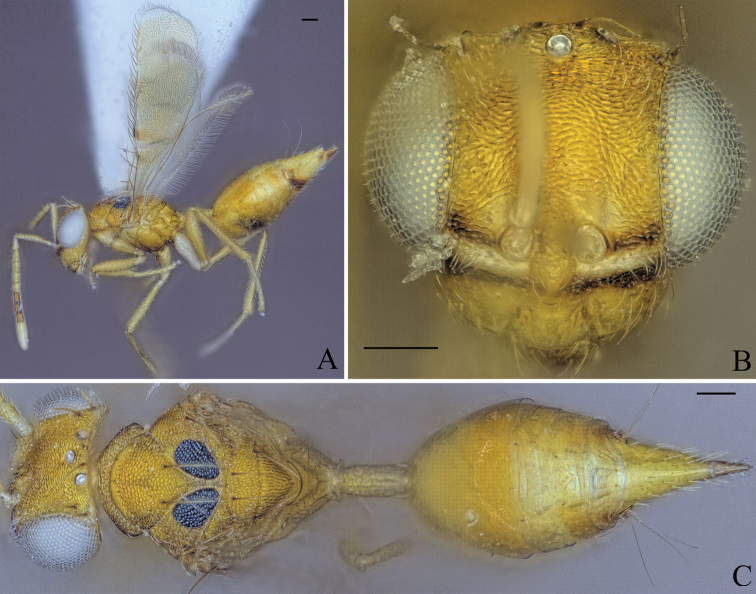
Holotype of *Diparanyani* sp. nov. **A** habitus in lateral view **B** face in frontal view **C** body in dorsal view. Scale bar: 100 µm.

**Female.** Body bright yellowish brown (Fig. 11A); face with two transverse stripes of dark brown coloration just at the level of toruli and at the level of the ventral margin of the eye, interrupted in interantennal area and supraclypeal area enclosing a stripe of pale white coloration (Fig. 11B); legs yellowish brown except for metacoxa white (Fig. 11A); macropterous, fore wing reaching gt7 (Fig. 11A); mesoscutellum large, mesosoma length 3.43–3.83× (3.43) mesoscutellum length (Fig. 11C); petiole medium to long, 1.78–2.05× (2.02) (specimens used for measurement: ZFMK-HYM-00037248, ZFMK-HYM-00037249, ZFMK-HYM-00037252, NHMUK013457234) as long as wide in dorsal view.

##### Description.

***Size*:** small to medium sized, body length 1696–2064 (2037) µm (specimens used for measurement: ZFMK-HYM-00037248, ZFMK-HYM-00037249, ZFMK-HYM-00037251, NHMUK013457234).

***Coloration*:** body bright yellowish brown (Fig. 11A); scape ventrally yellowish white, dorsally brown, pedicel and f1–4 yellowish brown, f5–7 brown, clava pale yellowish white (Fig. 11A); face with two transverse stripes of dark brown coloration just at the level of toruli and at the level of the ventral margin of the eye, interrupted in interantennal area and supraclypeal area enclosing a stripe of pale white coloration (Fig. 11B); mesoscutum with pair of black spots medially on lateral area (Fig. 11C); two infuscate spots at the upper edge of the fore wing, one at 1/3 of the length and the other one in the middle (Fig. 11A); legs yellowish brown except for metacoxa white (Fig. 11A); gt6 and gt7 with brown spots around cerci (Fig. 11A); tip of ovipositor sheath brown (Fig. 11A).

***Head*:** head round, 1.25–1.30× (1.30) wider than high (Fig. 11B); head except for lower face subreticulate (Fig. 11B); upper face laterally sparsely setose (Fig. 11B); lower face smooth and sparsely setose (Fig. 11B); distance of antennal insertion to eye short, 0.51–0.94× (0.94) torulus diameter (Fig. 11B); antennae close, toruli separated by 1.12–1.28× (1.28) torulus diameter (Fig. 11B); antennal formula: 11173 (Fig. 11A); shape of funicle segments changing: from f1 longer than wide to f7 ca. as long as wide (Fig. 11A); malar space 0.33–0.39× (0.33) eye height (Fig. 11A); POL 1.24–1.41× (1.38) OOL (Fig. 11C).

***Mesosoma*:** pronotum short and slim, 3.84–5.79× (5.79) as wide as long, reticulate (Fig. 11C); mesosoma robust to of medium breadth, head breadth 1.16–1.33× (1.23) mesoscutum breadth (Fig. 11C); mesonotum completely reticulate (Fig. 11C); mesoscutum with two pairs of bristles: one pair on median area anterior of notauli, reaching posterior edge of mesoscutum, one pair laterally on lateral area anterior of wing base (Fig. 11C); notauli converging slightly anterior of posterior margin of mesoscutum (Fig. 11C); mesoscutellum with two pairs of bristles: one pair anterio-medially and one pair laterally on frenal line (Fig. 11C); macropterous, fore wing reaching gt7, with large bristles along submarginal vein and smaller bristles along marginal and postmarginal vein on edge, stigmal vein very short, stigma rounded, uncus short and pointed (Fig. 11A); propodeum medially smooth, laterally transversely carinate to carinate on nucha (Fig. 11C).

***Metasoma*:** petiole medium to long, 1.78–2.05× (2.02) (specimens used for measurement: ZFMK-HYM-00037248, ZFMK-HYM-00037249, ZFMK-HYM-00037252, NHMUK013457234) as long as wide in dorsal view, costate-rugose, with lateral pair of large white setae visible in dorsal view (Fig. 11C); gaster medium, 1.20–1.29× (1.25) longer than mesosoma in dorsal view (Fig. 11C); gt1 covering ~ 1/3 of gaster, gt2–4 ca. equal in size, gt5 and 6 much smaller (Fig. 11C); gt7 and ovipositor sheath sparsely setose (Fig. 11A).

##### Remarks.

*Diparanyani* is very similar to *D.kakamegensis*. It differs from it in the following characters: *D.nyani* is macropterous and the mesoscutellum is larger relative to the mesosoma length, based on the results of the morphometric analysis (Fig. 3). The stripes across the face are similar in *D.maculata*, *D.reticulata* and *D.rodneymulleni*. *Diparanyani* differs from *D.maculata* in having a yellowish brown mesocoxa and petiole. *Diparanyani* differs from *D.rodneymulleni* in many characters: the body coloration, the length of the petiole and the body shape. *Diparanyani* differs from *D.reticulata* in having smooth gastral tergites while they are reticulated in *D.reticulata*.

##### Male.

Unknown.

##### Etymology.

Named after the word for monkey in the national language of Kenya, Swahili, because of the dorsal black dots and the mesoscutellum which resemble the face of a monkey.

##### Biology.

***Habitat*:** Leaf litter.

***Host*:** Unknown.

##### Distribution.

Kenya.

#### Dipara reticulata sp. nov.

Taxon classificationAnimaliaHymenopteraPteromalidae

9D533226-5296-5B9D-B228-5BE56D718974

http://zoobank.org/7ACD0DFB-0D61-437D-AA97-80FBF09E3540

Fig. 12A–C

##### Material examined.

***Holotype*** Kenya • 1 ♀; Kakamega Forest, Kenya; 00°14'6.1N, 34°52'9.2E; 1605 m a.s.l.; 28 Aug. 2007; Hita-Garcia, F. leg.; Winkler extraction; Transect 23; ZFMK-HYM-00040373. ***Paratypes*** Kenya • 1 ♀; Kakamega Forest, Kenya; 00°14'52.3N, 34°52'5.3E; 1607 m a.s.l.; 14 Aug. 2007; Hita-Garcia, F. leg.; Winkler extraction; Transect 18; ZFMK-HYM-00040374 • 2 ♀; Kakamega Forest, Kenya; 00°14'52.3N, 34°52'5.3E; 1607 m a.s.l.; 21 Aug. 2007; Hita-Garcia, F. leg.; Winkler extraction; Transect 18; NHMUK013457236; NMK: ZFMK-HYM-00040376.

##### Diagnosis.

**Figure 12.**
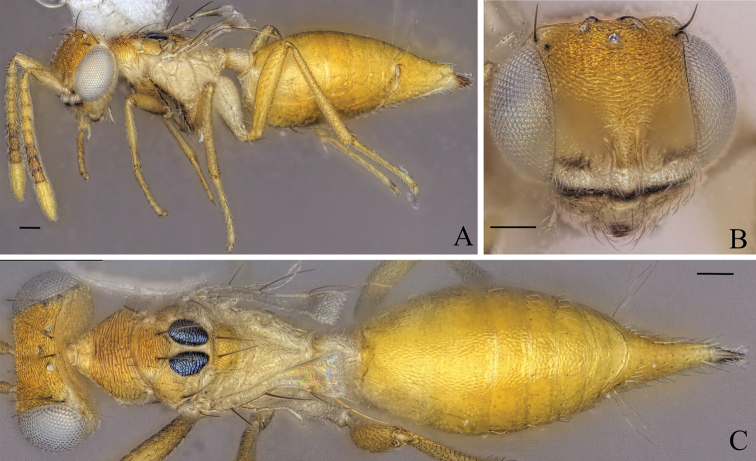
Holotype of *Diparareticulata* sp. nov. **A** habitus in lateral view **B** face in frontal view **C** body in dorsal view. Scale bar: 100 µm.

**Female.** Gastral tergites reticulate (Fig. 12C).

##### Description.

***Size*:** medium sized, body length 2303–2927 (2303) µm.

***Coloration*:** vertex and upper face brown to orangish brown, lower face yellowish brown (Fig. 12B); face with two transverse dark brown stripes just at the level of toruli and at the level of the ventral margin of the eye enclosing a stripe of white coloration, lower stripe darker than upper, upper stripe much fainter in interantennal area (Fig. 12C); scape, pedicel, first to fourth funicle segment (f1–f4) and clava yellowish brown (Fig. 12A); f5–f7 brown (Fig. 12A); pronotum and median area of mesoscutum brown to orangish brown (Fig. 12C); lateral area of mesoscutum and mesoscutellum yellowish brown (Fig. 12C); two black spots with metallic tint medially on lateral area of mesoscutum (Fig. 12C); procoxa, lower mesepisternum and anterior part of mesocoxa dark brown, rest of mesocoxa pale brown, metacoxa white with darker brown part anteriorly, rest of legs yellowish brown (Fig. 12A); mesosoma laterally, propodeum and petiole white (Fig. 12C); fore wing transparent with infuscation at tip (Fig. 12A); gaster yellowish brown (Fig. 12A); ovipositor sheath anteriorly white and posterior tip dark brown (Fig. 12A).

***Head*:** head oval, 1.35–1.42× (1.35) wider than high (Fig. 12B); head except for lower face strigate-reticulate, lower face reticulate and sparsely setose (Fig. 12B); distance of antennal insertion to eye long, 1.29–1.56× (1.34) torulus diameter (Fig. 12B); antennae mostly far apart, toruli separated by 1.31–1.58× (1.31) torulus diameter (Fig. 12B); antennal formula: 11173 (Fig. 12A); funicle segments ca. as long as wide (Fig. 12A); malar space 0.26–0.29× (0.27) eye height (Fig. 12A); POL 0.93–1.12× (0.93) OOL (Fig. 12C).

***Mesosoma*:** pronotum large and elongated, 1.78–2.38× (1.78) wider than long, substrigate, with two transverse rows of setae on posterior 1/2 (Fig. 12C); mesosoma mostly slender, head breadth 1.48–1.60× (1.53) mesoscutum breadth (Fig. 12C); notauli converging ca. at 1/2 the length of mesoscutum (Fig. 12C); median area of mesoscutum substrigate, black spots on lateral area strigate-reticulate, lateral area laterally reticulate (Fig. 12C); axillae, mesoscutellum, and frenum reticulate (Fig. 12C); mesoscutum with some small brown setae and two pairs of large bristles: one pair on median area just anterior of notauli, reaching posterior edge of mesoscutum, one pair laterally on lateral area anterior of wing base (Fig. 12C); axillae with some small brown setae (Fig. 12C); mesoscutellum with one pair of bristles anterio-medially and one pair of smaller setae laterally, anterior of frenal line, frenum much smaller than rest of mesoscutellum (Fig. 12C); brachypterous, tips truncated, fore wing reaching middle of petiole, three large black bristles along edge and one large brown bristle at the tip (Fig. 12A); propodeum medially rugulose and laterally transversely carinate transitioning to carinate on nucha (Fig. 12C).

***Metasoma*:** petiole short, 1.20–1.31× (1.24) wider than long, rugose, with four pairs small white setae laterally (Fig. 12C); gastral tergites reticulate (Fig. 12C); gaster medium, 1.47–1.56× (1.54) longer than mesosoma in dorsal view (Fig. 12C); gt1 covering ~1/3 of gaster, gt2–4 ca. equal in size, gt5–6 smaller (Fig. 12C); gt7 and ovipositor sheath sparsely setose (Fig. 12A).

##### Remarks.

*Diparareticulata* is similar to *D.kakamegensis*, *D.maculata*, *D.nyani*, and *D.rodneymulleni* in having transverse stripes across the face. *Diparareticulata* is different form all other *Dipara* species in having reticulated gastral tergites. In all other species the gastral tergites are smooth.

##### Male.

Unknown.

##### Etymology.

Named for the reticulated gastral tergites.

##### Biology.

***Habitat*:** Leaf litter.

***Host*:** Unknown.

##### Distribution.

Kenya.

#### Dipara rodneymulleni sp. nov.

Taxon classificationAnimaliaHymenopteraPteromalidae

3BC7918E-5F83-592B-B057-E4D9252EEC9E

http://zoobank.org/879AFBBB-0A9B-4F26-A577-183E31E05118

Fig. 13A–C

##### Material examined.

***Holotype*** Kenya • 1 ♀; Kakamega Forest, Kenya; 00°19'36N, 34°52'14.6E; 1570 m a.s.l.; 28 Jun. 2007; Hita-Garcia, F. leg.; Winkler extraction; Transect 6; ZFMK-HYM-00040369. ***Paratypes*** Kenya • 2 ♀; Kakamega Forest, Kenya; 00°23'6.2N, 34°33'37.8E; 1602 m a.s.l.; 16 Jul. 2007; Hita-Garcia, F. leg.; Winkler extraction; Transect 11; ZFMK-HYM-00040370; NHMUK013457235 • 1 ♀; Kakamega Forest, Kenya; 00°21'21.1N, 34°51'44.9E; 1632 m a.s.l.; 01 Aug. 2007; Hita-Garcia, F. leg.; Winkler extraction; Transect 16; NMK: ZFMK-HYM-00040372.

##### Diagnosis.

**Figure 13.**
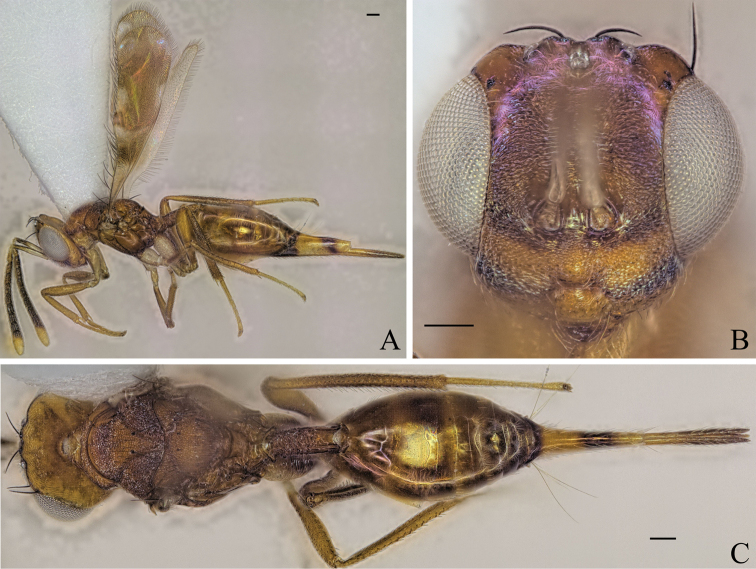
Holotype of *Dipararodneymulleni* sp. nov. **A** habitus in lateral view **B** face in frontal view **C** and body in dorsal view. Scale bar: 100 µm.

**Female.** Face with two dark brown stripes at the level of the ventral margin of the eye, interrupted in supraclypeal area, and at the level of the toruli (Fig. 13B); absence of black spots on median area of mesoscutum (Fig. 13C); petiole very long, 2.53–2.80× (2.79) longer than wide (Fig. 13C).

##### Description.

***Size*:** medium to large, body length 2718–3397 (3397) µm.

***Coloration*:** body brown (Fig. 13); distal quarter of scape and pedicel, all funicle segments and small proximal part of the first claval segment (c1) dark brown, rest of scape white, rest of pedicel and clava yellowish brown (Fig. 13A); face with two dark brown stripes at the level of the ventral margin of the eye, interrupted in supraclypeal area, and at the level of the toruli (Fig. 13B); fore leg yellowish brown (Fig. 13A); mid leg with coxa and trochanter white, rest brown (Fig. 13A); hind leg with coxa white with dark brown coloration on posterior part, tibia dark brown, rest brown (Fig. 13A); gt7 with dark brown coloration around cerci and on posterior 1/2, rest of gt7 yellowish brown (Fig. 13A); ovipositor sheath yellowish brown on anterior 1/2 and posterior tip dark brown (Fig. 13A).

***Head*:** head round, 1.22–1.29× (1.29) (specimens used for measurement: ZFMK-HYM-00040369, ZFMK-HYM-00040372, NHMUK013457235) wider than high (Fig. 13B); upper and lower face reticulate (Fig. 13B); vertex and interantennal area smooth (Fig. 13B); antennal scrobe substrigate (Fig. 13B); distance of antennal insertion to eye long, 1.49–1.76× (1.68) (specimens used for measurement: ZFMK-HYM-00040369, ZFMK-HYM-00040372, NHMUK013457235) torulus diameter (Fig. 13B); antennae close, toruli separated by 0.99–1.04× (0.99) (specimens used for measurement: ZFMK-HYM-00040369, ZFMK-HYM-00040372, NHMUK013457235) torulus diameter (Fig. 13B); antennal formula: 11173 (Fig. 13A); funicle segments longer than wide, getting shorter from f1–7 (Fig. 13A); malar space 0.30–0.32× (0.30) eye height (Fig. 13A); lower face and vertex sparsely setose (Fig. 13A); occipital margin with sharp edge (Fig. 13A); POL 1.47–1.74× (1.74) OOL (Fig. 13C).

***Mesosoma*:** pronotum short and slim, 3.29–3.81× (3.74) as wide as long, strigulate-reticulate, sparsely setose (Fig. 13C); mesosoma of medium breadth, head breadth 1.25–1.28× (1.25) (specimens used for measurement: ZFMK-HYM-00040369, ZFMK-HYM-00040370, NHMUK013457235) mesoscutum breadth (Fig. 13C); notauli not converging (Fig. 13C); mesoscutum reticulate, sparsely setose, with two pairs of bristles: one pair medially on median area anterior of notauli almost reaching axillae, one pair laterally on lateral area anterior of wing base (Fig. 13C); axillae reticulate (Fig. 13C); mesoscutellum anteriorly reticulate to carinulate posteriorly and on frenum, with two pairs of bristles: one pair anterio-medially and one pair laterally on frenal line, frenum almost as large as anterior part of mesoscutellum (Fig. 13C); macropterous, fore wing with large black bristles along submarginal vein and smaller bristles along marginal and postmarginal vein, mostly infuscate with some transparent patches, stigmal vein rather short, stigma thin, uncus short and pointed (Fig. 13A); propodeum medially smooth and laterally confused carinate (Fig. 13C); nucha carinate (Fig. 13C).

***Metasoma*:** petiole very long, 2.53–2.80× (2.79) longer than wide, with anterior 2/3 rugose and rest carinate, with lateral pair of large white setae visible in dorsal view (Fig. 13C); gaster medium, 1.46–1.53× (1.53) longer than mesosoma in dorsal view (Fig. 13C); gt1 covering ~1/3 of gaster, gt2–6 ca. equal in size (Fig. 13C); gt7 and ovipositor sheath slender and elongated, together ca. as long as rest of gaster, sparsely setose (Fig. 13C).

##### Remarks.

*Dipararodneymulleni* shares the stripes across the face with *D.maculata*, *D.nyani*, *D.kakamegensis*, and *D.reticulata* and but other than that has a completely different morphology and coloration. The most obvious characters to distinguish *D.rodneymulleni* are the very long petiole and the absence of black spots on the lateral areas of the mesoscutum.

**Male.** Unknown.

##### Etymology.

Named after professional skateboarder Rodney Mullen who revolutionized street skating like no other, reflecting the first author’s lifelong passion for skateboarding.

##### Biology.

***Habitat*:** Leaf litter.

***Host*:** Unknown.

##### Distribution.

Kenya.

#### Dipara sapphirus sp. nov.

Taxon classificationAnimaliaHymenopteraPteromalidae

126AD965-D123-529D-8C0B-5B9A03952783

http://zoobank.org/16FE1162-7E49-488A-A922-A84D432CA22B

Fig. 14A–C

##### Material examined.

***Holotype*** Kenya • 1 ♀; Kakamega Forest, Kenya; 00°13'15.5N, 34°53'24.7E; 1597 m a.s.l.; 23 Aug. 2007; Hita-Garcia, F. leg.; Winkler extraction; Transect 22; ZFMK-HYM-00040339. ***Paratypes*** Kenya • 1 ♀; Kakamega Forest, Kenya; 00°37'24.1N, 34°51'12E; 1585 m a.s.l.; 16 Aug. 2007; Hita-Garcia, F. leg.; Winkler extraction; Transect 10; ZFMK-HYM-00040340 • 7 ♀; Kakamega Forest, Kenya; 00°21'4.4N, 34°51'41.1E; 1602 m a.s.l.; 05 Jun. 2007; Hita-Garcia, F. leg.; Winkler extraction; Transect 2; ZFMK-HYM-00040341, ZFMK-HYM-00040353 to ZFMK-HYM-00040356; NMK: ZFMK-HYM-00040357, ZFMK-HYM-00040358 • 3 ♀; Kakamega Forest, Kenya; 00°27'0.9N, 34°50'52.9E; 1649 m a.s.l.; 10 Jul. 2007; Hita-Garcia, F. leg.; Winkler extraction; Transect 8; ZFMK-HYM-00040342; NHMUK013457227, NHMUK013457228 • 7 ♀; Kakamega Forest, Kenya; 00°21'7.9N, 34°52'2.6E; 1597 m a.s.l.; 09 Jul. 2007; Hita-Garcia, F. leg.; Winkler extraction; Transect 7; ZFMK-HYM-00040343, ZFMK-HYM-00040362, ZFMK-HYM-00040363; NMK: ZFMK-HYM-00040359 to ZFMK-HYM-00040361, ZFMK-HYM-00040366 • 1 ♀; Kakamega Forest, Kenya; 00°27'10.6N, 34°51'48.7E; 1676 m a.s.l.; 19 Jun. 2007; Hita-Garcia, F. leg.; Winkler extraction; Transect 4; ZFMK-HYM-00040344 • 3 ♀; Kakamega Forest, Kenya; 00°21'4.4N, 34°51'41.1E; 1602 m a.s.l.; 05 Jun. 2007; Hita-Garcia, F. leg.; Winkler extraction; Transect 2; ZFMK-HYM-00040345 to ZFMK-HYM-00040347 • 3 ♀; Kakamega Forest, Kenya; 00°19'49.9N, 34°52'16.1E; 1580 m a.s.l.; 01 Aug. 2007; Hita-Garcia, F. leg.; Winkler extraction; Transect 15; NHMUK013457229 to NHMUK013457231 • 1 ♀; Kakamega Forest, Kenya; 00°21'7.9N, 34°52'2.6E; 1597 m a.s.l.; 02 Jul. 2007; Hita-Garcia, F. leg.; Winkler extraction; Transect 7; ZFMK-HYM-00040364 • 1 ♀; Kakamega Forest, Kenya; 00°23'6.2N, 34°33'37.8E; 1602 m a.s.l.; 23 Jul. 2007; Hita-Garcia, F. leg.; Winkler extraction; Transect 11; ZFMK-HYM-00040365 • 1 ♀; Kakamega Forest, Kenya; 00°13'15.5N, 34°53'24.7E; 1597 m a.s.l.; 25 Aug. 2007; Hita-Garcia, F. leg.; Winkler extraction; Transect 22; ZFMK-HYM-00040367 • 1 ♀; Kakamega Forest, Kenya; 00°13'15.5N, 34°53'24.7E; 1597 m a.s.l.; 23 Aug. 2007; Hita-Garcia, F. leg.; Winkler extraction; Transect 22; ZFMK-HYM-00040368.

**Female** (specimens used for morphometric measurements: ZFMK-HYM-00040339 to ZFMK-HYM-00040343).

##### Diagnosis.

**Figure 14.**
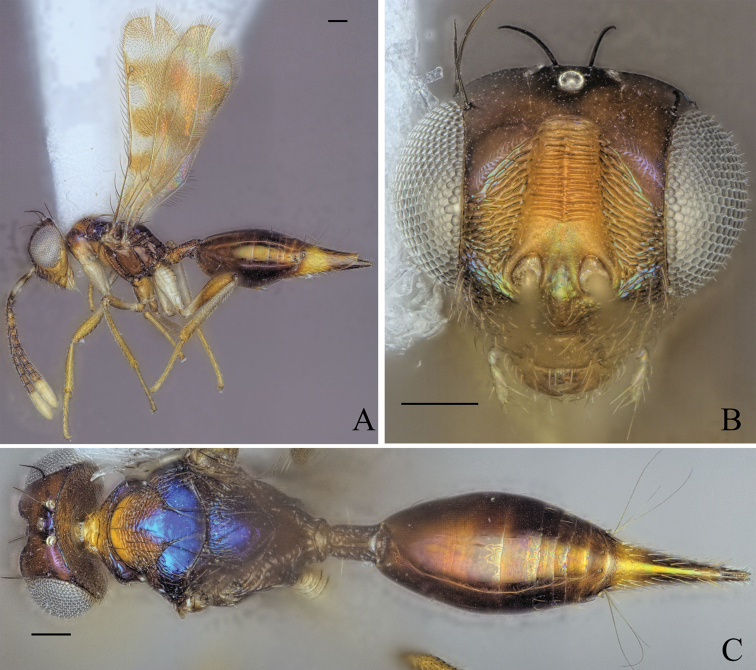
Holotype of *Diparasapphirus* sp. nov. **A** habitus in lateral view **B** face in frontal view **C** body in dorsal view. Scale bar: 100 µm.

Strong blue metallic tint on the following areas: vertex between ocelli, pronotum laterally, median area of mesoscutum posteriorly between notauli, lateral area of mesoscutum and mesoscutellum (Fig. 14C).

##### Description.

***Size*:** small to medium sized, body length 1667–2432 (2081) µm.

***Coloration*:** body dark brown (Fig. 14); scape proximally and distally dark brown, medially white, proximal 1/2 of pedicel dark brown, distal 1/2 white, funicle segments dark brown, clava pale yellowish white (Fig. 14A); strong blue metallic tint on the following areas: vertex between ocelli, pronotum laterally, median area of mesoscutum posteriorly between notauli, lateral area of mesoscutum and mesoscutellum (Fig. 14C); fore leg with coxa, trochanter and proximal 1/3 of femur white, rest yellowish brown (Fig. 14A); mid leg with proximal parts of femur and tibia yellowish white, rest yellowish brown (Fig. 14A); hind leg with distal 2/3 of femur brown, distal quarter of tibia and tarsus yellowish brown, rest white (Fig. 14A); anterior 1/2 of gt7 yellowish brown (Fig. 14A).

***Head*:** head round, 1.26–1.30× (1.29) wider than high (Fig. 14B); upper face strigate-reticulate (Fig. 14B); lower face reticulate and sparsely setose (Fig. 14B); antennal scrobe substrigate with deep groove (Fig. 14B); vertex and interantennal area smooth (Fig. 14B); distance of antennal insertion to eye short, 0.85–1.08× (1.00) torulus diameter (Fig. 14B); antennae close, separated by 1.07–1.26× (1.16) torulus diameter (Fig. 14B); antennal formula: 11173 (Fig. 14A); funicle segments ca. as long as wide (Fig. 14A); malar space 0.26–0.41× (0.26) eye height (Fig. 14A); POL 0.78–0.95× (0.95) OOL (Fig. 14C).

***Mesosoma*:** pronotum mostly short and slim, 3.30–3.60× (3.60) wider than long, medially and around posterior margin smooth, laterally reticulate, with some setae close to the posterior edge (Fig. 14C); mesosoma mostly robust, head breadth 1.13–1.22× (1.14) mesoscutum breadth (Fig. 14C); notauli not converging (Fig. 14C); mesoscutum with median area posteriorly between notauli smooth and rest strigate-reticulate, lateral area medially smooth and laterally reticulate, sparsely setose, with two pairs of larger bristles: one pair on median area just anterior of notauli, reaching posterior margin of mesoscutum, one pair laterally on lateral area anterior of wing base (Fig. 14C); axillae smooth and sparsely setose (Fig. 14C); mesoscutellum anteriorly reticulate to smooth posteriorly, with two pairs of bristles: one pair anterio-medially and one pair laterally on frenal line (Fig. 14C); macropterous, fore wing reaching gt7 with large bristles along submarginal vein and smaller bristles along marginal and postmarginal vein on edge, alternating infuscate and transparent, starting with infuscate at the tip, stigmal vein short, stigma round and large, uncus broad and rounded (Fig. 14A); propodeum medially smooth, laterally transversely carinate (Fig. 14C); nucha carinate (Fig. 14C).

***Metasoma*:** petiole short to medium, 1.31–1.67× (1.60) longer than wide in dorsal view, costate-rugose, with lateral pair of large white setae visible in dorsal view (Fig. 14C); gaster medium 1.37–1.53× (1.47) longer than mesosoma in dorsal view (Fig. 14C); gt1 covering ~ 1/2 of gaster, gt2 larger than gt3–6, gt3–6 ca. equal in size (Fig. 14C); gt7 and ovipositor sheath elongated and sparsely setose (Fig. 14A).

##### Remarks.

In body shape, *D.sapphirus* is similar to *D.lux* and *D.tenebra* but can be distinguished from all other *Dipara* species by having a very distinct blue metallic tint on the following body parts: vertex between ocelli, pronotum laterally, median area of mesoscutum posteriorly between notauli, lateral area of mesoscutum and mesoscutellum (Fig. 14C).

##### Male.

Unknown.

##### Etymology.

Named after sapphires for the blue metallic tint.

##### Biology.

***Habitat*:** Leaf litter.

***Host*:** Unknown.

##### Distribution.

Kenya.

#### Dipara tenebra sp. nov.

Taxon classificationAnimaliaHymenopteraPteromalidae

7A645A18-799B-5F08-A626-529E9D6A93AE

http://zoobank.org/90CA2ECF-B5A6-44E6-8CCF-80C6EF6A37F6

Fig. 15A–C

##### Material examined.

***Holotype*** Kenya • 1 ♀; Kakamega Forest, Kenya; 00°27'10.6N, 34°51'48.7E; 1676 m a.s.l.; 19 Jun. 2007; Hita-Garcia, F. leg.; Winkler extraction; Transect 4; ZFMK-HYM-00040377. ***Paratype*** Kenya • 1 ♀; Kakamega Forest, Kenya; 00°37'24.1N, 34°51'12E; 1585 m a.s.l.; 01 Aug. 2007; Hita-Garcia, F. leg.; Winkler extraction; Transect 10; ZFMK-HYM-00040378.

##### Diagnosis.

**Figure 15.**
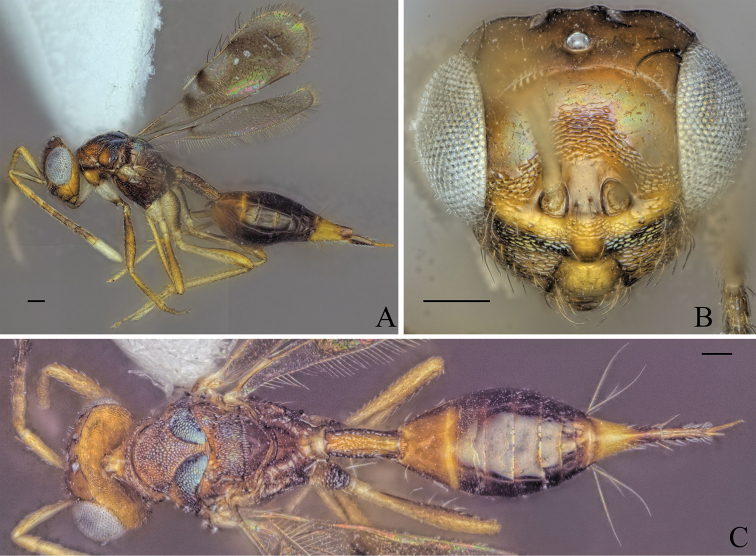
Holotype of *Diparatenebra* sp. nov. **A** habitus in lateral view **B** face in frontal view **C** body in dorsal view. Scale bar: 100 µm.

**Female.** Body brown to dark brown (Fig. 15); face with dark brown to black stripe from one eye to the other at the level of the ventral margin of the eye, interrupted in supraclypeal area (Fig. 15B); vertex smooth (Fig. 15B); petiole very long, 2.51–2.77× (2.51) longer than wide in dorsal view (Fig. 15C).

##### Description.

***Size*:** medium sized, body length 2293–2474 (2293) µm.

***Coloration*:** body brown to dark brown (Fig. 15); scape and f1 yellowish brown, pedicel, f23, and f7 yellowish brown to brown, f4–6 brown, clava white (Fig. 15A); face with dark brown to black stripe from one eye to the other at the level of the ventral margin of the eye, interrupted in supraclypeal area (Fig. 15B); mesoscutum with two black spots medially on lateral area (Fig. 15C); fore leg with distal tip of coxa brown, rest of coxa white, trochanter and femur brown, tibia and tarsus yellowish brown (Fig. 15A); mid leg with coxa and trochanter white and rest yellowish brown (Fig. 15A); hind leg with anterior part of coxa, trochanter and anterior part of femur white, posterior part of coxa dark brown to black, rest of hind leg yellowish brown (Fig. 15A); gt1 brown, anterior 2/3 of gt7 yellowish brown, rest of gaster dark brown (Fig. 15A).

***Head*:** head oval, 1.33–1.37× (1.33) wider than high (Fig. 15B); upper face next to toruli reticulate, rest smooth (Fig. 15B); lower face reticulate, sparsely setose (Fig. 15B); interantennal area smooth, antennal scrobe strigate-reticulate (Fig. 15B); vertex smooth (Fig. 15B); distance of antennal insertion to eye short, 0.66–0.76× (0.66) torulus diameter (Fig. 15B); antennae close, toruli separated by 1.17–1.32× (1.17) torulus diameter (Fig. 15B); antennal formula: 11173 (Fig. 15A); funicle segments getting shorter from f1 to f7, f1 much longer than wide, f7 ca. as wide as long (Fig. 15A); malar space 0.37–0.39× (0.37) eye height (Fig. 15A); POL 0.89–0.96× (0.89) OOL (Fig. 15C).

***Mesosoma*:** pronotum of medium length, 3.11–3.12× (3.11) wider than long, substrigate, with row of setae close to the posterior edge (Fig. 15C); mesosoma robust, head breadth 1.15–1.18× (1.15) mesoscutum breadth (Fig. 15C); mesoscutum reticulate, with two pairs of bristles: one pair on median area anterior of notauli, one pair laterally on lateral area anterior to wing base (Fig. 15C); notauli converging at posterior margin of mesoscutum (Fig. 15C); axillae reticulate (Fig. 15C); mesoscutellum anteriorly reticulate, frenum smooth, with two pairs of bristles: one pair anterio-medially, one pair laterally anterior of frenal line (Fig. 15C); macropterous, fore wing reaching gt7, with larger bristles along marginal and postmarginal vein on edge of forewing, with dense brush of setae at proximal end of marginal vein, with large areas of infuscation, stigmal vein very short, stigma large and rounded, uncus short and pointed (Fig. 15A); propodeum medially smooth and laterally transversely carinate (Fig. 15C); nucha carinate (Fig. 15C).

***Metasoma*:** petiole very long, 2.51–2.77× (2.51) longer than wide in dorsal view, costate-rugose, with lateral pair of large setae visible in dorsal view (Fig. 15C); gaster medium, 1.20–1.24× (1.20) longer than mesosoma in dorsal view (Fig. 15C); gt1 covering ~ 1/3 of gaster (Fig. 15C); gt7 and ovipositor sheath sparsely setose (Fig. 15A).

##### Remarks.

*Diparatenebra* is similar to *D.corona*, *D.lux*, *D.machadoi*, *D.striata*, *D.tigrina*, *and D.turneri* and in having one dark brown to black stripe across the face. *Diparatenebra* is different from *D.machadoi* in having distinct notauli, which are lacking in *D.machadoi*. It differs from *D.corona*, *D.striata*, *D.tigrina*, *and D.turneri* in having a very long petiole. *Diparatenebra* and *D.lux* are very similar in body shape and differ in their body coloration which is much darker in *D.tenebra* and in the surface sculpture of the head. They share the otherwise unique character of having a dense brush of setae close to the proximal end of the marginal vein on the fore wing.

##### Male.

Unknown.

##### Etymology.

Named after the Latin word *tenebra* for darkness, in contrast to *D.lux* which looks very similar but is much lighter in coloration.

##### Biology.

***Habitat*:** Leaf litter.

***Host*:** Unknown.

##### Distribution.

Kenya.

#### Dipara tigrina sp. nov.

Taxon classificationAnimaliaHymenopteraPteromalidae

0B2CC181-A12E-58BC-B741-7D73EFA31770

http://zoobank.org/E816ADB7-A279-4978-81DE-7F24CCF38422

Fig. 16A–D

##### Material examined.

***Holotype*** Kenya • 1 ♀; Kakamega Forest, Kenya; 00°21'4.4N, 34°51'41.1E; 1602 m a.s.l.; 05 Jun. 2007; Hita-Garcia, F. leg.; Winkler extraction; Transect 2; ZFMK-HYM-00040383.

**Figure 16.**
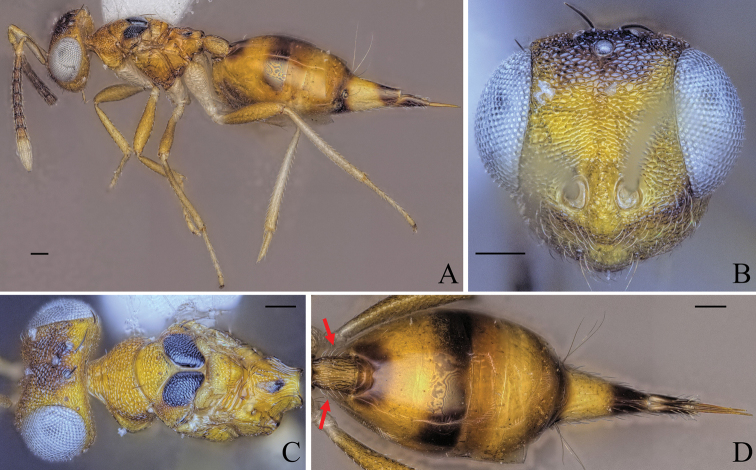
Holotype of *Diparatigrina* sp. nov. **A** habitus in lateral view **B** face in frontal view **C** head and mesosoma in dorsal view **D** metasoma in dorsal view; red arrow: setae laterally on the petiole. Scale bar: 100 µm.

**Diagnosis. Female.** Propodeum laterally smooth, medially distinctly subcarinate with reticulation between carinae, carinae extending to nucha (Fig. 16C); petiole with at least six pairs of small white setae laterally (Fig. 16D).

##### Description.

***Size*:** medium sized, body length 2329 µm.

***Coloration*:** body yellowish brown to brown (Fig. 16); dorsal part of scape brown, ventral part of scape white, pedicel yellowish brown, funicle segments dark brown, clava white (Fig. 16A); face yellowish brown, with two brown spots at the ventral margin of the eye (Fig. 16B); vertex dark brown (Fig. 16B); mesoscutum with two large black spots medially on lateral area (Fig. 16C); fore and mid leg with coxa white, rest yellowish brown (Fig. 16A); hind leg with coxa, trochanter and proximal 1/2 of tibia white, rest yellowish brown (Fig. 16A); two broad dark brown stripes on gt1, one directly posterior to petiole and one at posterior edge (Fig. 16D); gt6 dark brown (Fig. 16D); gt7 around cerci and posterior 1/3 dark brown, rest yellowish brown (Fig. 16A); ovipositor sheath dark brown (Fig. 16A).

***Head*:** head round, 1.24× wider than high, entirely reticulate (Fig. 16B); lower face sparsely setose (Fig. 16B); distance of antennal insertion to eye short, 0.66 torulus diameter (Fig. 16B); antennae close, toruli separated by 1.31× torulus diameter (Fig. 16B); antennal formula: 11173 (Fig. 16A); funicle segments getting shorter from f1 to f7, f1 slightly longer than wide and f7 as wide as long (Fig. 16A); malar space 0.34× eye height (Fig. 16A); POL 1.03× OOL (Fig. 16C).

***Mesosoma*:** pronotum large and elongated, 1.95× as wide as long, reticulate, with two rows of small setae close to posterior margin (Fig. 16C); mesosoma of medium breadth, head breadth 1.46× mesoscutum breadth (Fig. 16C); notauli converging at 1/2 the length of mesoscutum (Fig. 16C); mesoscutum reticulate, with two pairs of bristles: one pair anterio-medially on median area anterior of notauli one pair laterally on lateral area anterior of wing base (Fig. 16C); axillae reticulate (Fig. 16C); mesoscutellum anteriorly reticulate, frenum porcate, with two pairs of bristles: one pair anterio-medially, one pair laterally just anterior of frenal line (Fig. 16C); brachypterous, fore wing reaching anterior edge of propodeum or shorter (Fig. 16C); propodeum laterally smooth, medially distinctly subcarinate with reticulated pattern between the carinae, carinae extending to nucha (Fig. 16C).

***Metasoma*:** petiole short, 1.37× as long as wide in dorsal view, with at least six pairs of small white setae laterally visible in dorsal view, subcarinate (Fig. 16D), similar to propodeum sculpture (Fig. 16C); gaster medium, 1.51× longer than mesosoma in dorsal view (Fig. 16D); gt1 covering ~ 1/3 of gaster (Fig. 16D); gt7 and ovipositor sheath sparsely setose (Fig. 16A).

##### Remarks.

*Diparatigrina* is similar to *D.corona*, *D.lux*, *D.machadoi*, *D.striata*, *D.tenebra*, *and D.turneri* in having one dark brown to black stripe across the face. It differs from *D.corona*, *D.lux*, *D.machadoi*, *D.tenebra*, and *D.turneri* in the propodeum sculpture. The propodeum sculpture is similar in *D.punctulata* and *D.striata*. They show a very distinct surface sculpture with a striated subcarinate pattern extending to the nucha. *Diparatigrina* differs from *D.punctulata* and *D.striata* in having more setae laterally on the propodeum and in having a reticulated pattern medially between the carinae on the propodeum.

The only available specimen of this species has an irregular black spot on the propodeum. This spot is considered an aberration and thus is not part of the species description.

**Male.** Unknown.

##### Etymology.

Named after the Latin adjective *tigrinus* for the tiger-like stripes on the gaster.

##### Biology.

***Habitat*:** Leaf litter.

***Host*:** Unknown.

##### Distribution.

Kenya.

#### Dipara albomaculata

Taxon classificationAnimaliaHymenopteraPteromalidae

(Hedqvist, 1963)

49558FC0-85DB-5178-97CA-03334CD5A442

Fig. 17A, B

Afrolelaps albomaculata Hedqvist 1963: 49–50.
Grahamisia albomaculata Hedqvist 1969: 185.
Dipara albomaculata Desjardins 2007: 42, 46.

##### Material examined.

***Paratype*** Angola • 1 ♀; Mabete, Caungula; 20. Jul. 1962; A. de Barros Machado leg.; NHMUK013455574.

##### Other material.

Kenya • 5 ♀; Kakamega Forest, Kenya; 00°22'43.7N, 34°41'57.3E; 1452 m a.s.l.; 25 Aug. 2008; Hita-Garcia, F. leg.; Winkler extraction; Transect 35; ZFMK-HYM-00040386 to ZFMK-HYM-00040390.

##### Diagnosis.

**Figure 17.**
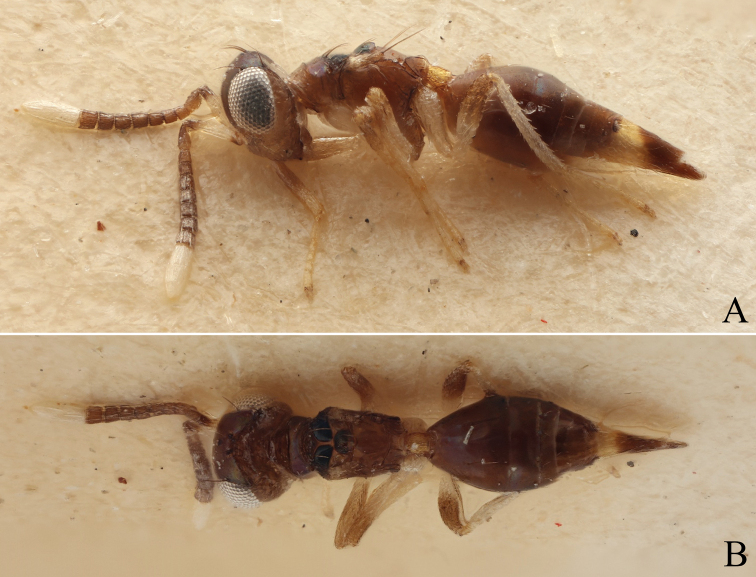
Paratype of *Diparaalbomaculata* (Hedqvist, 1963) **A** habitus in lateral view **B** body in dorsal view.

**Female.** Body brown to dark brown (Fig. 17); vertex smooth (Fig. 17B); clava white (Fig. 17A); lateral area of mesoscutum completely black (Fig. 17B); pro- and metacoxa white (Fig. 17A); propodeum completely smooth (Fig. 17B); gt1 with a pair of large bristles dorso-anteriorly (Fig. 17B).

##### Remarks.

The holotype of *D.albomaculata* is supposed to be stored at the MDLA but we were unable to get in contact with the museum and thus the holotype could not be located and examined. Two paratypes are stored at the BMNH and one of them was examined.

*Diparaalbomaculata* is similar to *D.andreabalzerae*, *D.fastigata*, *D.nigroscutellata* and *D.saetosa* in having a black mesoscutellum while the general body coloration is not black. It differs from *D.andreabalzerae* and *D.fastigata* in having a pair of bristles dorso-anteriorly on the gt1. It differs from *D.nigroscutellata* in the general body coloration, which is much darker and in the coloration of the lateral area of the mesoscutum. In *D.albomaculata* the lateral area is completely black and *D.nigroscutellata* has a small yellowish brown area on its most lateral part. The differences to *D.saetosa* can be found in the smooth vertex and the white pro- and metacoxa.

*Diparaalbomaculata* is similar to *D.straminea* in sharing the bristles on the gt1 and the propodeum sculpture. It differs from *D.straminea* in having a black mesoscutellum.

Additional specimens from this species were found in the Kakamega Forest in Kenya and the distribution is updated accordingly.

##### Distribution.

Angola; Kenya.

#### Dipara machadoi

Taxon classificationAnimaliaHymenopteraPteromalidae

(Hedqvist, 1971)

FAA15383-8544-5766-8168-CBD51E85CD41

Diparomorpha machadoi Hedqvist 1971: 55–59.
Dipara machadoi Desjardins 2007: 42, 48–50.

##### Diagnosis.

**Female.** Notauli absent.

##### Remarks.

The holotype of *D.machadoi* is supposed to be stored at the MDLA but we were unable to get in contact with the museum and thus the holotype could not be located and examined. Based on the original description by Hedqvist (1971)*D.machadoi* differs from all other Afrotropical *Dipara* species in having no notauli.

#### Dipara maculata

Taxon classificationAnimaliaHymenopteraPteromalidae

(Hedqvist, 1963)

75BAAECF-E639-5DB9-AC5D-6C363D9CE830

Afrolelaps maculata Hedqvist 1963: 47–49.
Grahamisia maculata Hedqvist 1969: 185.
Dipara maculata Desjardins 2007: 42, 46.

##### Diagnosis.

**Female.** Face with two transverse stripes of dark brown coloration just at the level of toruli and at the level of the ventral margin of the eye, enclosing a stripe of pale yellowish white coloration; mesocoxa and petiole white.

##### Remarks.

The holotype of *D.maculata* is supposed to be stored at the MDLA but we were unable to get in contact with the museum and thus the holotype could not be located and examined. Based on the original description by Hedqvist (1963) it is similar to *D.kakamegensis*, *D.nyani*, and *D.rodneymulleni* in having two transverse stripes on the face. It differs from *D.rodneymulleni* in having a much shorter petiole. In contrast to *D.kakamegensis* and *D.nyani*, *D.maculata* has a white petiole and mesocoxa.

#### Dipara nigrita

Taxon classificationAnimaliaHymenopteraPteromalidae

Hedqvist, 1969

79C160FF-E690-585D-A3D1-9281B7ADC9D1

Fig. 18A–D , 19A–C

Dipara nigrita Hedqvist, 1969: 195.

##### Material examined.

***Holotype*** Democratic Republic Of Congo • 1 ♀; Mount Kabobo, Terr. Albertville, Hte. Kiymbi; 1700 m a.s.l; Oct. 1958; N. Leleup leg.; “Humus en forêt”; RMCA ENT 000017982.

##### Other material.

Kenya • 1 ♀; Kakamega Forest, Kenya; 00°14'22.9N, 34°51'21E; 1594 m a.s.l.; 24 Jul. 2007; Hita-Garcia, F. leg.; Winkler extraction; Transect 12; ZFMK-HYM-00040384 • 1 ♀; Kakamega Forest, Kenya; 00°19'49.9N, 34°52'16.1E; 1580 m a.s.l.; 01 Aug. 2007; Hita-Garcia, F. leg.; Winkler extraction; Transect 15; ZFMK-HYM-00040385.

##### Diagnosis.

**Figure 18.**
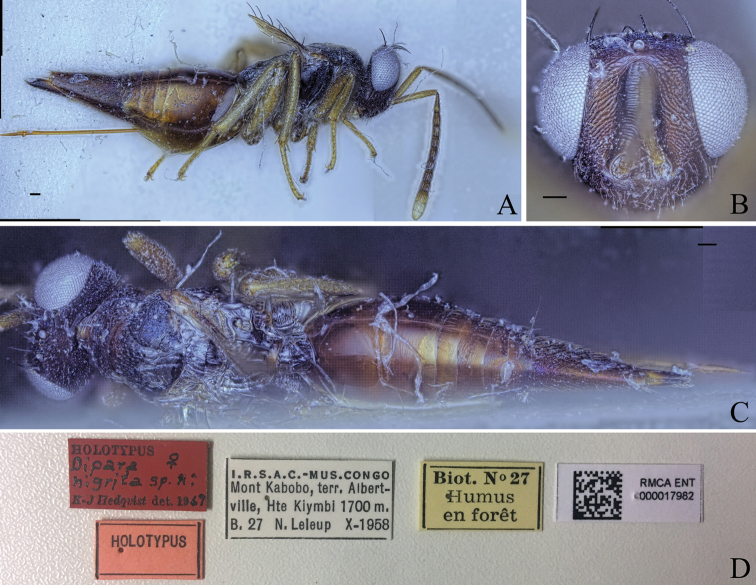
Holotype of *Diparanigrita* Hedqvist, 1969 **A** habitus in lateral view **B** face in frontal view **C** body in dorsal view **D** labels. Scale bar: 100 µm.

**Figure 19.**
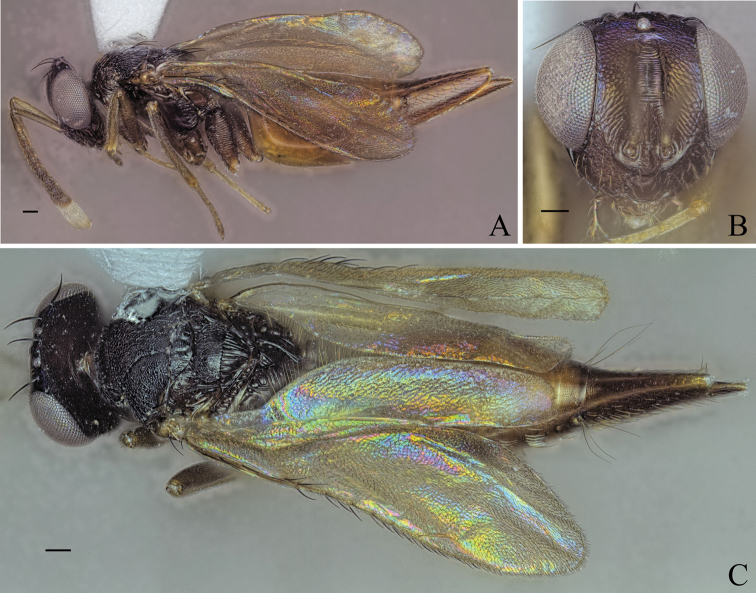
Macropterous specimen of *Diparanigrita* Hedqvist, 1969 from the Kakamega Forest in Kenya **A** habitus in lateral view **B** face in frontal view **C** body in dorsal view. Scale bar: 100 µm.

**Female.** Head and mesosoma black, coxae dark brown (Figs 18, 19).

##### Variation.

*Diparanigrita* was originally described as brachypterous (Fig. 18) by Hedqvist (1969). In the examined material from Kenya, we found specimens that we consider to be the macropterous form of this species (Fig. 19). Differences in the wing form within Diparinae are reported from several other species (Bouček 1988; Mitroiu 2019) and the slight differences found between the macropterous forms and the brachypterous holotype were not enough to justify describing the macropterous form as a new species. Those differences were found in the color of the first claval segment. It can vary from light brown to white.

Macropterous individuals have fully developed wings with the fore wings reaching the gt7 (Fig. 19). Brachypterous individuals show much shorter wings with the fore wings reaching approximately the posterior margin of the petiole (Fig. 18).

##### Remarks.

*Diparanigrita* is the only species which shows a completely black coloration of the head and mesosoma. Darker specimens of *D.albomaculata* sometimes have a partly very dark brown to black head and mesosoma but never completely black. Additionally, the coxa of *D.albomaculata* are white in contrast to the dark brown coxa of *D.nigrita*.

Additional specimens from the species were found in the Kakamega Forest in Kenya and the distribution is updated accordingly.

##### Distribution.

Democratic Republic of Congo; Kenya.

#### Dipara pallida

Taxon classificationAnimaliaHymenopteraPteromalidae

(Hedqvist, 1969)

97B3572C-B125-55BB-B3FA-85EC38DC8442

Fig. 20A–B

Pondia pallida Hedqvist 1969: 198–199.
Dipara pallida Desjardins 2007: 42.

##### Material examined.

***Holotype*** South Africa • 1 ♀; Port St. John, Pondoland; Jan. 1924; R.E. Turner leg.; NHMUK013455580.

##### Diagnosis.

**Figure 20.**
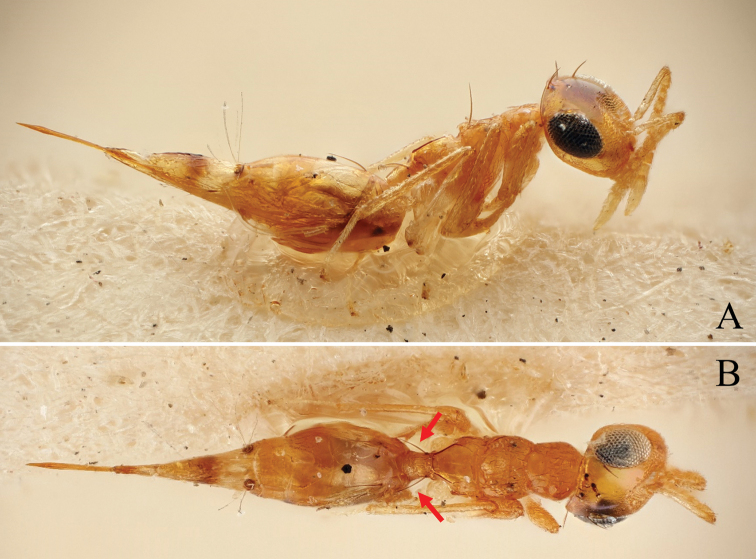
Holotype of *Diparapallida* (Hedqvist, 1969) **A** habitus in lateral view **B** face in frontal view **C** body in dorsal view; red arrows: long lateral bristles on the petiole.

**Female.** Vertex and propodeum smooth (Fig. 20B); petiole with long bristle anterio-laterally, reaching gt1 (Fig. 20B).

##### Remarks.

*Diparapallida* is similar to *D.punctulata* in having a large bristle anterio-laterally on the petiole. They differ in the surface sculpture of the vertex and the propodeum.

**Figure 21.**
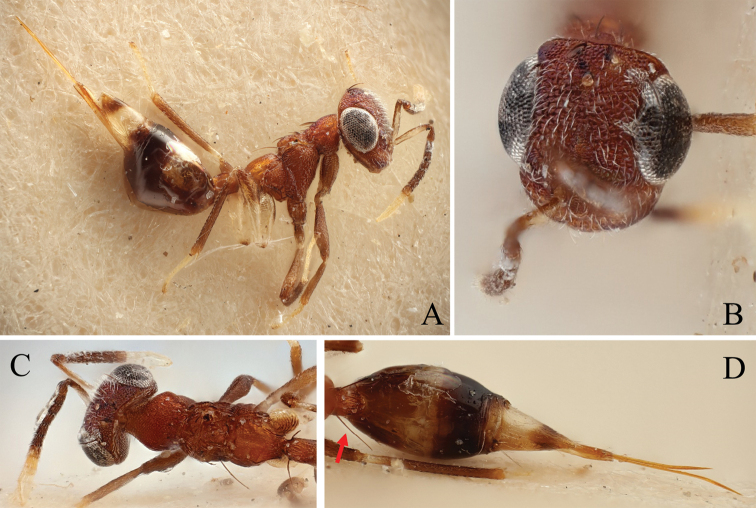
Holotype of *Diparapunctulata* (Hedqvist, 1969) **A** habitus in lateral view **B** face in frontal view **C** head and mesosoma in dorsal view **D** metasoma in dorsal view; red arrow: long lateral bristles on the petiole.

#### Dipara punctulata

Taxon classificationAnimaliaHymenopteraPteromalidae

(Hedqvist, 1969)

816FDF70-816C-51EE-A193-789CE9AD42F1

Fig. 21A–D

Pondia punctulata Hedqvist 1969: 197–198.
Dipara punctulata Desjardins 2007: 42.

##### Material examined.

***Holotype*** South Africa • 1 ♀; Port St. John, Pondoland; Jan. 1924; R.E. Turner leg.; NHMUK013455579.

##### Diagnosis.

**Female.** Vertex reticulate (Fig. 21B); propodeum subcarinate (Fig. 21C); petiole with long bristle anterio-laterally, reaching gt1 (Fig. 21D).

##### Remarks.

*Diparapunctulata* is similar to *D.pallida* in having a large bristle anterio-laterally on the petiole. They differ in the surface sculpture of the vertex and the propodeum.

#### Dipara saetosa

Taxon classificationAnimaliaHymenopteraPteromalidae

(Delucchi, 1962)

1F52C5E1-A55F-5BCB-A4D0-8134ADE9AED0

Fig. 22A–D

Grahamisia saetosa Delucchi 1962: 379.
Dipara saetosa Desjardins 2007: 42, 46.

##### Material examined.

***Holotype*** Tanzania • 1 ♀; Tanganyika Terr., Mt. Oldeani, versant Est; 2350–2500 m a.s.l.; 6.–9. Jun. 1957; RMCA ENT 000017989.

##### Diagnosis.

**Figure 22.**
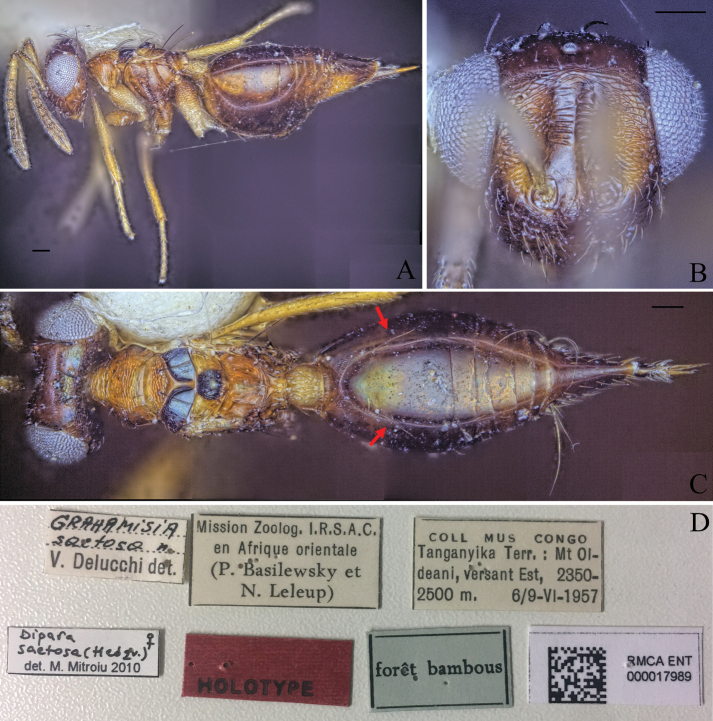
Holotype of *Diparasaetosa* (Delucchi, 1962) **A** habitus in lateral view **B** face in frontal view **C** body in dorsal view **D** labels; red arrows: dorsal bristles on gt1. Scale bar: 100 µm.

**Female.** Vertex reticulate between ocelli, rest smooth (Fig. 22C); clava dark brown (Fig. 22A); lateral area of mesoscutum completely black (Fig. 22C); mesoscutellum black (Fig. 22C); pro- and metacoxa with proximal 1/3 brown and rest yellowish brown (Fig. 22A); gt1 with a pair of large bristles dorso-anteriorly (Fig. 22C).

##### Remarks.

*Diparasaetosa* is similar to *D.albomaculata*, *D.nigroscutellata*, and *D.straminea* in having a pair of large bristles dorso-anteriorly on the gt1. It differs from *D.straminea* in having a black mesoscutellum. In contrast to *D.nigroscutellata* the lateral area of the mesoscutum is completely black. Differences to *D.albomaculata* can be found in the reticulation between the ocelli and the coloration of the clava and the pro- and metacoxa.

#### Dipara straminea

Taxon classificationAnimaliaHymenopteraPteromalidae

(Hedqvist, 1969)

043F4D40-531C-53E2-B101-48D7B33A93FD

Fig. 23A–D

Grahamisia straminea Hedqvist 1969: 187–188.
Dipara straminea Desjardins 2007: 42, 46.

##### Material examined.

***Holotype*** Democratic Republic Of Congo • 1 ♀; Kivu, Terr. Mwenga, S.-O. Tombwe, Luiko; 2100 m a.s.l.; Jan. 1952; N. Leleup leg.; “Récolté dans l’humus”; RMCA ENT 000017981.

##### Diagnosis.

**Figure 23.**
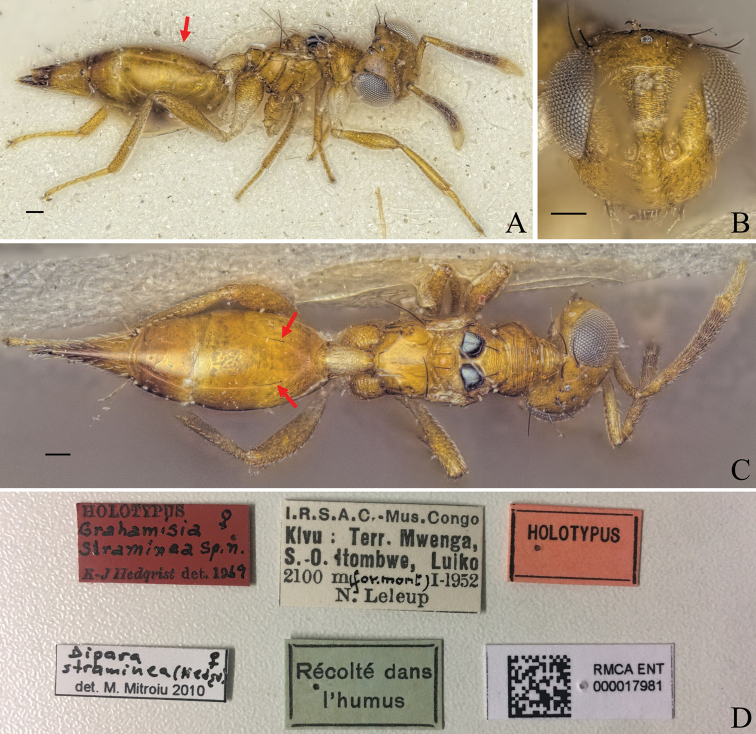
Holotype of *Diparastraminea* (Hedqvist, 1969) **A** habitus in lateral view **B** face in frontal view **C** body in dorsal view **D** labels; red arrows: dorsal bristles on gt1. Scale bar: 100 µm.

**Female.** Mesoscutellum yellowish brown (Fig. 23C); gt1 with a pair of large bristles dorso-anteriorly (Fig. 23A, C).

##### Remarks.

*Diparastraminea* is similar to *D.albomaculata*, *D.nigroscutellata*, and *D.saetosa* in having a pair of large bristles dorso-anteriorly on the gt1. It differs from them by having a yellowish brown mesoscutellum.

#### Dipara striata

Taxon classificationAnimaliaHymenopteraPteromalidae

(Hedqvist, 1969)

469A9403-E1A5-53BD-A659-05F08B804172

Fig. 24A–C

Grahamisia striata Hedqvist 1969: 188.
Dipara striata Desjardins 2007: 42, 46.

##### Material examined.

***Holotype*** South Africa • 1 ♀; Cape Province, Somerset East; 1.–26. Jan. 1931; R.E. Turner leg.; NHMUK013455578.

##### Diagnosis.

**Figure 24.**
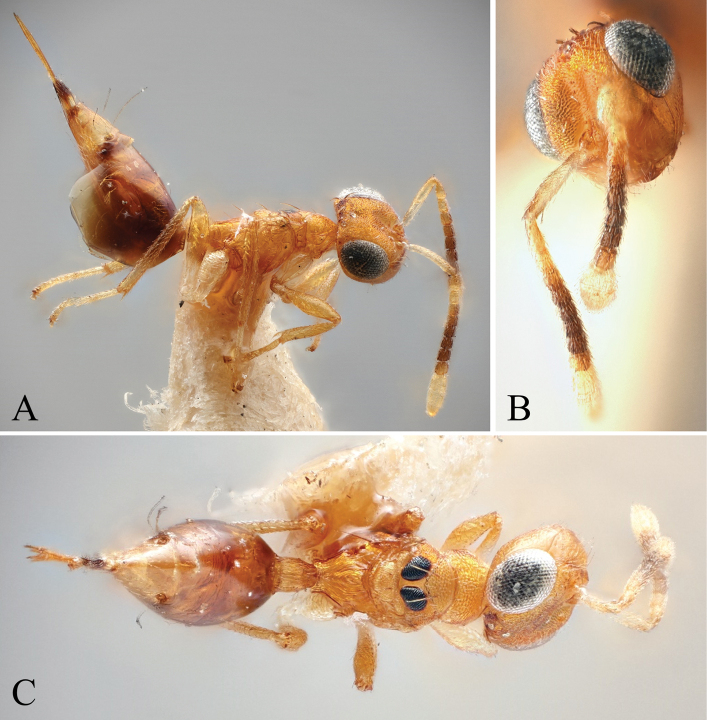
Holotype of *Diparastriata* (Hedqvist, 1969) **A** habitus in lateral view **B** face in frontal view **C** body in dorsal view.

**Female.** Propodeum laterally smooth, medially distinctly subcarinate, carinae extending to nucha (Fig. 24C); petiole with three pairs of small setae laterally (Fig. 24C).

##### Remarks.

*Diparastriata* is similar to *D.corona*, *D.lux*, *D.machadoi*, *D.tenebra*, *D.tigrina*, and *D.turneri* in having one dark brown to black stripe across the face. It differs from *D.corona*, *D.lux*, *D.machadoi*, *D.tenebra*, and *D.turneri* in the propodeum sculpture. The propodeum sculpture is similar in *D.punctulata* and *D.tigrina*. They show a very distinct striated subcarinate pattern extending to the nucha. *Diparastriata* differs from *D.punctulata* in lacking a large bristle anterio-laterally on the petiole. *Diparastriata* differs from *D.tigrina* in having less setae laterally on the petiole and in lacking reticulation on the propodeum.

#### Dipara turneri

Taxon classificationAnimaliaHymenopteraPteromalidae

Hedqvist, 1969

E611C1A7-5DDE-5CD1-B7F1-FCDB507306E6

Fig. 25A–C

Dipara turneri Hedqvist 1969: 193–194.

##### Material examined.

***Holotype*** South Africa • 1 ♀; Port St. John, Pondoland; 6.–25. Feb. 1924; R.E. Turner leg.; NHMUK013455576.

**Figure 25.**
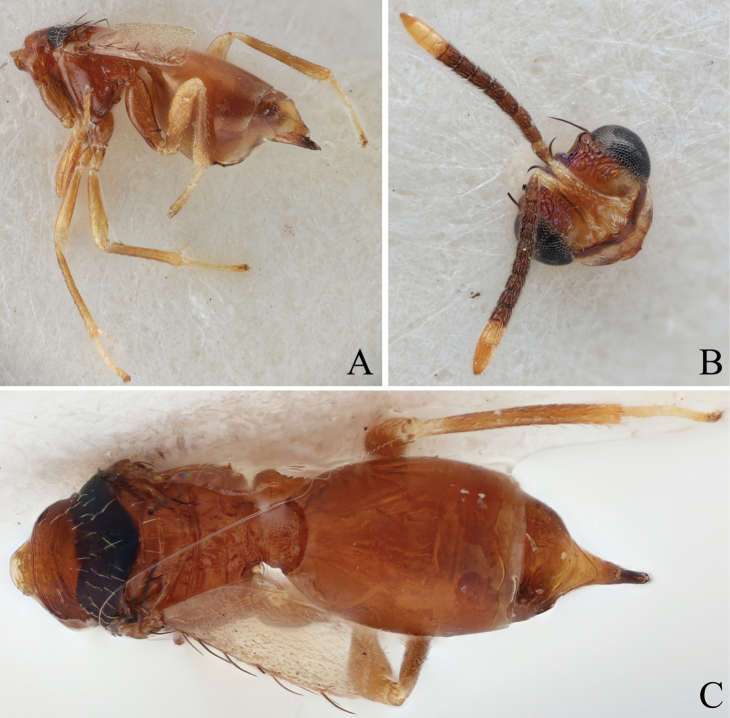
Holotype of *Diparaturneri*Hedqvist 1969**A** habitus in lateral view **B** face in frontal view **C** body in dorsal view.

**Diagnosis. Female.** Broad dark brown stripe across head from one eye to the other below toruli (Fig. 25B); median and lateral area of mesoscutum with distinct transverse broad black stripe (Fig. 25C); brachypterous, fore wing reaching slightly beyond petiole (Fig. 25A); petiole slightly wider than long (Fig. 25C).

##### Remarks.

*Diparaturneri* is similar to *D.corona* in having a distinct transverse broad black stripe on the median and lateral area of the mesoscutum. In other not completely black species the black spots on the mesoscutum are restricted to the lateral area.

*Diparaturneri* differs from *D.corona* in the wing form and in the petiole shape. The petiole is longer than wide in *D.corona* and wider than long in *D.turneri*.

## Discussion

Our results confirm that there is still a lot of undiscovered diversity within Microhymenoptera and the genus *Dipara* in particular (Desjardins 2007; Sharkey 2007; Aguiar et al. 2013; Forbes et al. 2018). Desjardins (2007) stated that there are “possibly hundreds of undescribed species” of *Dipara* left. We can support this statement based on the number of new *Dipara* species found only in the leaf litter in the small forest fragment Kakamega Forest, which more than doubled the number of known species from the Afrotropical mainland.

Some of the species descriptions in this study are based on so far unparalleled series of *Dipara* specimens. While most of the previously described *Dipara* species are known only from the holotype or just a few specimens, *D.kakamegensis*, for example, is described from 108 specimens and *D.nigroscutellata* from 86 specimens. These large series allowed for an advanced insight into intraspecific variation of *Dipara* species. The characters used for the species descriptions and diagnoses were found to be consistent among the large series, which gave us some confidence in delimiting species using the same characters in species with less specimens available. Our insights reveal that in most cases Afrotropical *Dipara* species seem to be reliably distinguishable by comparatively simple morphological characters of females like color patterns, surface sculpture or the number and position of setae or bristles. However, the intraspecific variation of the wing form of Diparinae females (Bouček 1988; Desjardins 2007; Mitroiu 2019) can pose a challenge for species delimitations, including those in this study. We decided to list wing related characters in the diagnoses and key but to always add additional non-wing characters.

While shedding more light on the species diversity of *Dipara* their biology remains largely unknown. All specimens were found in the leaf litter confirming that this might be their preferred habitat (Desjardins 2007). Reduced wings in females, which is found in 17 out of 22 Afrotropical species, can most likely be regarded as an adaptation to their ground-dwelling lifestyle and their search for hosts in the leaf litter or the soil. To gain more information about the hosts of *Dipara* more studies focusing on their biology would be needed.

Adding information on the biology, taxonomy, and distribution of species, is a crucial task. We still have only very limited knowledge on the biodiversity on this planet. We are aware, though, that we are facing presumably unprecedented biodiversity loss, especially through habitat destruction, and that this is one of the most pressing problems of our time (Steffen et al. 2015). The tropics including the Afrotropics are especially under threat because of ongoing deforestation and changes in land use, while also being biodiversity hotspots (Brooks et al. 2002). For example, the Kakamega Forest is the last large continuous forest in Kenya (Holstein 2015) and an officially protected area, but it is still under threat of habitat destruction (KIFCON 1994; Bleher et al. 2006; Lung and Schaab 2006; Müller and Mburu 2009). Studying the diversity of parasitoid wasps or other species-rich, abundant but understudied taxa can be a decisive tool for highlighting their importance for ecosystems, for conservation efforts and for understanding the evolution of the insects’ megadiversification. This contribution to our knowledge on the genus *Dipara* might serve as a small but valuable addition to the overwhelming picture of the biodiversity of the Afrotropics.

## Supplementary Material

XML Treatment for Dipara

XML Treatment for Dipara andreabalzerae

XML Treatment for Dipara corona

XML Treatment for Dipara fastigata

XML Treatment for Dipara kakamegensis

XML Treatment for Dipara lux

XML Treatment for Dipara nigroscutellata

XML Treatment for Dipara nyani

XML Treatment for Dipara reticulata

XML Treatment for Dipara rodneymulleni

XML Treatment for Dipara sapphirus

XML Treatment for Dipara tenebra

XML Treatment for Dipara tigrina

XML Treatment for Dipara albomaculata

XML Treatment for Dipara machadoi

XML Treatment for Dipara maculata

XML Treatment for Dipara nigrita

XML Treatment for Dipara pallida

XML Treatment for Dipara punctulata

XML Treatment for Dipara saetosa

XML Treatment for Dipara straminea

XML Treatment for Dipara striata

XML Treatment for Dipara turneri

## Supplementary materials

Supplementary material 1 Table S1 Data type Xslx file. Explanation note Measurements for the morphometric analysis of *Diparakakamegensis* sp. nov. and *Diparanyani* sp. nov. See Table 1 for definitions of the characters. https://binary.pensoft.net/file/604748This dataset is made available under the Open Database License (http://opendatacommons.org/licenses/odbl/1.0/). The Open Database License (ODbL) is a license agreement intended to allow users to freely share, modify, and use this Dataset while maintaining this same freedom for others, provided that the original source and author(s) are credited.Christoph Braun, Ralph S. Peters

Supplementary material 2 Table S2 Data type Xslx file. Explanation note Morphometric measurements for the descriptions of the newly described *Dipara* species. See Table 1 for definitions of the characters. N: measurement not available. https://binary.pensoft.net/file/604749This dataset is made available under the Open Database License (http://opendatacommons.org/licenses/odbl/1.0/). The Open Database License (ODbL) is a license agreement intended to allow users to freely share, modify, and use this Dataset while maintaining this same freedom for others, provided that the original source and author(s) are credited.Christoph Braun, Ralph S. Peters

Supplementary material 3 Table S3 Data type Xslx file. Explanation note Shape ratios for the descriptions of the newly described *Dipara* species. See Table 1 for definitions of the characters and Table 1 for definitions of the characters and Table 2 for definitions of the shape categories. N: measurement not available. https://binary.pensoft.net/file/604750This dataset is made available under the Open Database License (http://opendatacommons.org/licenses/odbl/1.0/). The Open Database License (ODbL) is a license agreement intended to allow users to freely share, modify, and use this Dataset while maintaining this same freedom for others, provided that the original source and author(s) are credited.Christoph Braun, Ralph S. Peters

## References

[B1] Aguiar AP Deans AR Engel MS Forshage M Huber JT Jennings JT Johnson NF Lelej AS Longino JT Lohrmann V Mikó I Ohl M Rasmussen C Taeger A Yu DSK (2013) Order Hymenoptera. Zootaxa 3703(1): 51–62. 10.11646/zootaxa.3703.1.12

[B2] Althof A (2005) Human Impact on Flora and Vegetation of Kakamega Forest, Kenya – Structure, distribution and disturbance of plant communities in an East African rainforest. PhD Thesis. Universität Koblenz-Landau, Koblenz-Landau, Germany.

[B3] Ashmead WH (1901) HymenopteraParasitica. Fauna Hawaii 1(3): 277–364.

[B4] Baur H (2015) Pushing the limits – two new species of *Pteromalus* (Hymenoptera, Chalcidoidea, Pteromalidae) from Central Europe with remarkable morphology. ZooKeys 514: 43–72. 10.3897/zookeys.514.9910 PMC4525024 26261432

[B5] Baur H Leuenberger C (2011) Analysis of ratios in multivariate morphometry. Systematic Biology 60(6): 813–825. 10.1093/sysbio/syr061 21828084 PMC3193766

[B6] Baur H Leuenberger C (2020, November 6) Multivariate Ratio Analysis (MRA): R-scripts and tutorials for calculating Shape PCA, Ratio Spectra and LDA Ratio Extractor (Version 1.05). Zenodo. 10.5281/zenodo.4250142

[B7] Baur H Kranz-Baltensperger Y Cruaud A Rasplus JY Timokhov AV Gokhman VE (2014) Morphometric analysis and taxonomic revision of *Anisopteromalus* Ruschka (Hymenoptera: Chalcidoidea: Pteromalidae) – an integrative approach. Systematic Entomology 39: 691–709. 10.1111/syen.12081 26074661 PMC4459240

[B8] BIOTA [BIOdiversity Monitoring Transect Analysis in Africa] (2010) BIOTA AFRICA. https://www.biota-africa.org/

[B9] Bleher B Uster D Bergsdorf T (2006) Assessment of Threat Status and Management Effectiveness in Kakamega Forest, Kenya. Biodiversity and Conservation 15(4): 1159–1177. doi: 10.1007/s10531-004-3509-3

[B10] Bouček Z (1988) Australasian Chalcidoidea (Hymenoptera): A biosystematic revision of genera of fourteen families, with a reclassification of species. C.A.B. International, Wallingford, 832 pp.

[B11] Brooks TM Mittermeier RA Mittermeier CG Da Fonseca GA Rylands AB Konstant WR Flick P Pilgrim J Oldfield S Magin G Hilton‐Taylor C (2002) Habitat loss and extinction in the hotspots of biodiversity. Conservation biology 16(4): 909–923. 10.1046/j.1523-1739.2002.00530.x

[B12] Clausnitzer V (2005) An updated checklist of the dragonflies (Odonata) of the Kakamega Forest, Kenya. Journal of East African Natural History 94(2): 239–246. 10.2982/0012-8317(2005)94[239:aucotd]2.0.co;2

[B13] Delucchi V (1962) Résultats scientifiques des missions zoologiques de l’I.R.S.A.C. en Afrique orientale (P. Basilewsky et N. Leleup, 1957), 81. HymenopteraChalcidoidea. Annales du Musée Royal de l’Afrique Centrale (Série in 8ø) Sciences Zoologique 110: 363–392.

[B14] Desjardins CA (2007) Phylogenetics and classification of the world genera of Diparinae (Hymenoptera: Pteromalidae). Zootaxa 1647(1): 1–88. 10.11646/zootaxa.1647.1.1

[B15] Domenichini G (1953) Studio sulla morfologia dell‘addome degli HymenopteraChalcidoidea. Bolletino die Zoologia Agraria e Bachicoltura 19: 183–297.

[B16] Forbes AA Bagley RK Beer MA Hippee AC Widmayer HA (2018) Quantifying the unquantifiable: why Hymenoptera, not Coleoptera, is the most speciose animal order. BMC Ecology 18(1): 1–11. 10.1186/s12898-018-0176-x 30001194 PMC6042248

[B17] Förster A (1856) Hymenopterologische Studien. 2. Chalcidiae und Proctotrupii. Aachen, 152 pp. 10.5962/bhl.title.8795

[B18] Gebiola M Monti MM Johnson RC Woolley JB Hunter MS Giorgini M Pedata PA (2017) A revision of the *Encarsiapergandiella* species complex (Hymenoptera: Aphelinidae) shows cryptic diversity in parasitoids of whitefly pests. Systematic Entomology 42: 31–59. 10.1111/syen.12187

[B19] Gibson GAP (1997) Morphology and terminology. In: Gibson GAP Huber JT Woolley JB (Eds) Annotated keys to the genera of Nearctic Chalcidoidea (Hymenoptera).NRC Research Press, Ottawa, 16–44.

[B20] Girault AA (1913) New genera and species of chalcidoid Hymenoptera in the South Australia Museum. Transactions of the Royal Society of South Australia 37: 67–115.

[B21] Girault AA (1915) Australian HymenopteraChalcidoidea – VI Supplement. Memoirs of the Queensland Museum 3: 313–346.

[B22] Girault AA (1915) Australian HymenopteraChalcidoidea – VIII. The family Miscogasteridae with descriptions of genera and species. Memoirs of the Queensland Museum 4: 185–202.

[B23] Girault AA (1927) Notes on and descriptions of chalcid wasps (Chalcididae) in the South Australian Museum. Records of the South Australian Museum 3: 309–338.

[B24] Girault AA (1933) Some beauties inhabitant not of the boudoirs of commerce but of nature’s bosom – new insects. Girault, Brisbane, 2 pp.

[B25] Graham MWRDV (1969) The Pteromalidae of North-Western Europe (Hymenoptera: Chalcidoidea). Bulletin of the British museum (Natural history) Entomology, Supplement 16: 1–908.

[B26] Harris RA (1979) A Glossary of Surface Sculpturing. Occasional Papers in Entomology 28: 1–31.

[B27] Hedqvist KJ (1963) New Diparini from Angola (Hym. Chalcidoidea). Publicaçoes Cultuarias da Companhia de Diamantes de Angola 63: 47–51.

[B28] Hedqvist KJ (1969) New genera and species of Diparini with notes on the tribe (Hym., Chalcidoidea). Entomologisk Tidskrift 90(3/4): 174–202.

[B29] Hedqvist KJ (1971) A new genus and species of Diparinae from Angola (Hym., Chalcidoidea, Pteromalidae). Publicaçoes Cultuarias da Companhia de Diamantes de Angola 84: 55–59.

[B30] Heraty JM Burks RA Cruaud A Gibson GAP Liljeblad J Munro J Rasplus JY Delvare G Jansta P Gumovsky A Huber JT Woolley JB Krogmann L Heydon S Polaszek A Schmidt S Darling DC Gates MW Mottern J Murray E Dal Molin A Triapitsyn S Baur H Pinto JD Van Noort S George J Yoder M (2013) A phylogenetic analysis of the megadiverse Chalcidoidea (Hymenoptera). Cladistics 29(5): 466–542. 10.1111/cla.12006 34798768

[B31] Hita-Garcia F Wiesel E Fischer G. (2013) The ants of Kenya (Hymenoptera: Formicidae) – faunal overview, first species checklist, bibliography, accounts for all genera, and discussion on taxonomy and zoogeography. Journal of East African Natural History 101(2): 127–222. 10.2982/028.101.0201

[B32] Holstein J (Ed) (2015) A Field Guide to insects and allies of the Kakamega Forest National Reserve. BIOTA Field Guide, Stuttgart, 292 pp.

[B33] Kieffer JJ (1906) Description de nouveaux Hyménoptères. Annales de la Société Scientifique de Bruxelles 30: 113–178.

[B34] Kieffer JJ Marshall TA (1904) Proctotrypidae. Species des Hyménoptères d’Europe et d’Algérie 9(85): 1–64.

[B35] KIFCON (1994) Kakamega Forest: The official guide. Kenya indigenous Forest Conservation Programme, Nairobi.

[B36] Kokwaro JO (1988) Conservation status of the Kakamega Forest in Kenya: the easternmost relic of the equatorial rain forests of Africa. Monographs in Systematic Botany 25: 471–489.

[B37] Kühne L (2008) Butterflies and moth diversity of the Kakamega Forest (Kenya). Brandenburgische Universitätsdruckerei, Potsdam-Babelsberg, 203 pp.

[B38] László Z Baur H Tóthmérész B (2013) Multivariate ratio analysis reveals *Trigonoderuspedicellaris* Thomson (Hymenoptera, Chalcidoidea, Pteromalidae) as a valid species. Systematic Entomology 38: 753–762. 10.1111/syen.12026

[B39] Mercet RG (1927) Nota sobre Lelapinos (Hym. Chalc.). Eos. Revista Española di Entomologia. Madrid 3: 49–63.

[B40] Mitroiu MD (2011) Diversity of the Afrotropical Pteromalidae (Hymenoptera): a preliminary assessment. Young Researchers 2011 / PhD Students, Young Scientists and Pedagogues Conference Proceedings (ISBN: 978-80-8094-946-4): 158–167.

[B41] Mitroiu MD (2019) Revision of *Netomocera* Bouček (Hymenoptera: Chalcidoidea: Pteromalidae), excluding the Oriental species. European Journal of Taxonomy (568). 10.5852/ejt.2019.568

[B42] Noyes JS (2019) Universal Chalcidoidea Database. World Wide Web electronic publication. http://www.nhm.ac.uk/chalcidoids

[B43] Peters RS Niehuis O Gunkel S Bläser M Mayer C Podsiadlowski L Kozlov A Donath A van Noort S Liu S Zhou X Misof B Heraty J Krogmann L (2018) Transcriptome sequence-based phylogeny of chalcidoid wasps (Hymenoptera: Chalcidoidea) reveals a history of rapid radiations, convergence, and evolutionary success. Molecular phylogenetics and evolution 120: 286–296. 10.1016/j.ympev.2017.12.005 29247847

[B44] Ross SRJ Hita-Garcia F Fischer G Peters MK (2018) Selective logging intensity in an East African rain forest predicts reductions in ant diversity. Biotropica 50(5): 768–778. 10.1111/btp.12569

[B45] Sharkey MJ (2007) Phylogeny and classification of Hymenoptera. Zootaxa 1668(1): 521–548. 10.11646/zootaxa.1668.1.25

[B46] Steffen W Richardson K Rockström J Cornell SE Fetzer I Bennett EM Biggs R Carpenter SR De Vries W De Wit CA Folke C (2015) Planetary boundaries: Guiding human development on a changing planet. Science 347(6223). doi: 10.1126/science.1259855 25592418

[B47] Thomson CG (1876) Hymenoptera Scandinaviae. Tom. IV. Pteromalus (Svederus). Lundae, 259 pp.

[B48] Walker F (1833) Monographia Chalciditum. Entomological Magazine 1: 367–384.

[B49] Yoder MJ Mikó I Seltmann KC Bertone MA Deans AR (2010) A Gross Anatomy Ontology for Hymenoptera. PLoS ONE 5(12): e15991. 10.1371/journal.pone.0015991 PMC3012123 21209921

